# Surfactant-enhanced DNA accessibility to nuclease accelerates phenotypic β-lactam antibiotic susceptibility testing of *Neisseria gonorrhoeae*

**DOI:** 10.1371/journal.pbio.3000651

**Published:** 2020-03-19

**Authors:** Emily S. Savela, Nathan G. Schoepp, Matthew M. Cooper, Justin C. Rolando, Jeffrey D. Klausner, Olusegun O. Soge, Rustem F. Ismagilov

**Affiliations:** 1 Division of Biology and Biological Engineering, California Institute of Technology, Pasadena, California, United States of America; 2 Division of Chemistry and Chemical Engineering, California Institute of Technology, Pasadena, California, United States of America; 3 David Geffen School of Medicine, Division of Infectious Disease, University of California Los Angeles, Los Angeles, California, United States of America; 4 Neisseria Reference Laboratory, Department of Global Health, University of Washington, Seattle, Washington, United States of America; Universitat zu Koln, GERMANY

## Abstract

Rapid antibiotic susceptibility testing (AST) for *Neisseria gonorrhoeae* (*Ng*) is critically needed to counter widespread antibiotic resistance. Detection of nucleic acids in genotypic AST can be rapid, but it has not been successful for β-lactams (the largest antibiotic class used to treat *Ng*). Rapid phenotypic AST for *Ng* is challenged by the pathogen’s slow doubling time and the lack of methods to quickly quantify the pathogen’s response to β-lactams. Here, we asked two questions: (1) Is it possible to use nucleic acid quantification to measure the β-lactam susceptibility phenotype of *Ng* very rapidly, using antibiotic-exposure times much shorter than the 1- to 2-h doubling time of *Ng*? (2) Would such short-term antibiotic exposures predict the antibiotic resistance profile of *Ng* measured by plate growth assays over multiple days? To answer these questions, we devised an innovative approach for performing a rapid phenotypic AST that measures DNA accessibility to exogenous nucleases after exposure to β-lactams (termed nuclease-accessibility AST [nuc-aAST]). We showed that DNA in antibiotic-susceptible cells has increased accessibility upon exposure to β-lactams and that a judiciously chosen surfactant permeabilized the outer membrane and enhanced this effect. We tested penicillin, cefixime, and ceftriaxone and found good agreement between the results of the nuc-aAST after 15–30 min of antibiotic exposure and the results of the gold-standard culture-based AST measured over days. These results provide a new pathway toward developing a critically needed phenotypic AST for *Ng* and additional global-health threats.

## Abstract 2

This study presents a new approach for testing antibiotic-susceptibility in the slow-growing human pathogen Neisseria gonorrhoeae, based on measuring accessibility of pathogen nucleic acids to extracellular reagents. Surprisingly, the beta-lactam susceptibility phenotype was revealed with antibiotic exposures shorter than cell division.

## Introduction

Gonorrhea, caused by *N*. *gonorrhoeae* (*Ng*), is the second most common notifiable sexually transmitted infection (STI) in the United States [1] and the third most common STI globally. Gonorrhea affects 86,900,000 people each year worldwide [2]. Untreated *Ng* infections can lead to pelvic inflammatory disease, infertility, ectopic pregnancy, and neonatal blindness [3] and have a significant financial burden on healthcare systems [4]. Antibiotic resistance in *Ng* emerged quickly and continues to spread unchecked because there is no rapid antibiotic susceptibility test (AST) to guide treatment. The Centers for Disease Control and Prevention (CDC) estimates that almost half (550,000) of the 1.14 million new *Ng* infections reported are antibiotic resistant [5]. Lacking a rapid AST, clinicians are limited to making empiric prescriptions as recommended by the CDC [6] or World Health Organization (WHO) [7]. When resistance to a particular antibiotic exceeds 5%, treatment guidelines are updated and the recommended treatment protocol is escalated to the next line of antibiotic [8,9]. As a result, *Ng* strains continue to evolve resistance, even to the last-line treatment (dual treatment with azithromycin [AZM]/ceftriaxone [CRO]) [10–12]. The global prevalence and spread of resistant *Ng* infections has led the CDC to place *Ng* in its highest (“urgent”) category of antimicrobial-resistant pathogen threats [13] and WHO to label *Ng* as a high-priority pathogen [14]. Despite the threat of untreatable *Ng* [15] and an international call for rapid diagnostics [16–18], no phenotypic AST currently exists that can be performed rapidly enough for the point of care (POC).

Successful and timely treatment of *Ng* infections while still considering antibiotic stewardship requires two sequential steps to be performed at the POC. First, an identification (ID) test is run on the patient’s sample (typically urine or swab) to confirm that the patient is infected with *Ng*. Then, an AST must be run on the sample to determine whether the infecting strain of *Ng* is susceptible to the available antibiotics, so that the correct treatment can be prescribed. The health crisis associated with antibiotic-resistant infections is internationally recognized [19], and substantial efforts (both academic [20–22] and commercial [23,24]) are making great progress toward shortening the time required to identify *Ng* infections. However, there is no published path toward development of a rapid phenotypic AST for *Ng*, especially for β-lactam antibiotics. Thus, even with swift diagnosis of an *Ng* infection, prescription of the correct antibiotics at the POC will remain bottlenecked by the lack of a rapid AST.

AST methods are either genotypic or phenotypic. Genotypic methods predict resistance by screening for the presence of known resistance genes, whereas phenotypic methods determine susceptibly and resistance by directly measuring an organism’s response to an antibiotic. Rapid genotypic methods exist for select antibiotic classes such as quinolones [25,26], but the diverse mechanisms of resistance present in *Ng* would require highly multiplexed assays for most other antibiotic classes [27,28], including β-lactams [29,30], which are the largest class of antibiotics for *Ng*. For example, hundreds of β-lactamase genes are known [31], and new resistance genes continue to emerge, making it challenging to design a comprehensive genotypic AST, even for a single organism. Only phenotypic AST methods provide the ability to directly detect resistance and susceptibility, regardless of the antibiotic’s mechanism of action. The current gold-standard AST for *Ng* is agar dilution, a phenotypic method that takes many days and is only performed in a small number of reference laboratories [32]. Efforts have been made to shorten the total assay time of culture-based techniques [33–35], but these methods still rely on multiple cell divisions and thus require many hours because of the slow doubling time (1–2 h) of *Ng*. The doubling time of *Ng* is impacted by many factors, including pH [36], temperature [37], initial cell concentration, media, and isolate [38]. Although differences in conditions are straightforward to control in experiments with clinical isolates, the effects of variable growth time will be much greater when considering the unknown composition in clinical samples, which can have great variability, particularly in bacterial load and pH.

A phenotypic AST rapid enough for the POC would be paradigm-shifting for *Ng* [39] because it would provide the correct timely treatment of infections, significantly reduce disease burden, and improve global surveillance efforts [40–42]. Until a POC diagnostic is developed for *Ng*, empiric prescribing of the last-line dual antibiotic therapy of AZM/CRO will likely continue, as it has in the US over the last 5 y [43]. Likewise, if informed antibiotic prescriptions cannot be made, resistance will continue to spread, at which point no currently available antibiotics will be recommended for treatment of *Ng*. Importantly, a rapid, phenotypic AST would greatly increase treatment options because if clinicians know which antibiotics will be efficacious for each infection, they can once again treat with antibiotics that are not prescribed in the current (empiric-based) system because of the risk of resistance. For example, even though cefixime (CFM) is no longer used as a first-line therapy for *Ng*, up to 95% of infections in the US are still susceptible to CFM [1,44]. Similarly, up to 77% of *Ng* infections are susceptible to tetracycline (TET) [1]. Therefore, having a POC AST could enable clinicians to once again safely prescribe CFM and other antibiotics [45]. Several recent cases of *Ng* infections resistant to AZM [46,47], or the currently recommended combination of AZM/CRO[10,12], were detected after treatment was administered, highlighting the critical need for faster diagnostics.

For an *Ng* AST to inform treatment decisions at the POC, the total assay time to determine phenotypic susceptibility must be greatly decreased [48–50]. Quantification of pathogen-specific nucleic acid (NA) concentrations has shown major promise for the rapid determination of susceptibility phenotype [51–54]. These methods rely on comparing the NA concentrations of control and antibiotic-treated samples and thus work well for rapidly dividing organisms and for antibiotics that directly affect NA replication. NA-based phenotypic AST methods also benefit from the high sensitivity of NA amplification, and fast isothermal amplification techniques have led to short total assay times [51]. For example, by measuring the concentration of *Escherichia coli* DNA, we have shown that the antibiotic-exposure step for phenotypic AST can be shortened to 15 min [55]. We also were able to achieve a phenotypic AST with a 10-min antibiotic-exposure time in *Ng* by measuring changes in RNA concentration after exposure to ciprofloxacin (CIP), which directly inhibits DNA replication and downstream translation [56]. However, for antibiotics that do not impact DNA replication or gene expression on short timescales, such as β-lactams, these NA-based AST techniques have proven difficult; the fastest published method for *Ng* still requires 4 h of β-lactam exposure [57]. Importantly, of the antibiotics prescribed for *Ng*, only one, CIP [56], has been demonstrated to be compatible with this existing NA-based approach.

In this work, we asked two questions. (1) Is it possible to use NA quantification to measure β-lactam susceptibility phenotype of *Ng* very rapidly, using antibiotic-exposure times (15–30 min) much shorter than the doubling time of *Ng* (1–2 h)? (2) Would such short-term antibiotic exposures predict the antibiotic resistance profile of *Ng* measured by plate growth assays over multiple days? To answer these questions, here we describe an innovation that enables a rapid, NA-based phenotypic AST for β-lactams, the largest class of antibiotics used to treat *Ng*. We hypothesized that cell wall damage caused by exposure to β-lactams could be exploited to determine phenotypic susceptibility faster than cell division. Our method, termed nuclease-accessibility AST (nuc-aAST), measures the accessibility of intracellular *Ng* DNA to exogenously added nucleases after a short antibiotic exposure. We also wished to test whether the total time of the assay could be further decreased by including an enhancement step, defined as a condition that would lead to greater differences in DNA accessibility between resistant and susceptible samples.

We chose to validate this proof-of-concept nuc-aAST using three β-lactams, penicillin (PEN), CFM, and CRO. Each of these three antibiotics represent first-line treatments at different points in the history of *Ng* treatment [58,59]. Additionally, CRO, in combination with AZM, is the current recommended (and last-line) treatment for *Ng*. Determining susceptibility to CRO is thus relevant not only for treatment but for surveillance efforts. Clinical urine samples were chosen (and urine was chosen as the matrix for contrived samples) because urine is one of the primary sample types used for *Ng* diagnosis, especially in males [6,59]. We chose to test only categorically susceptible or resistant isolates, based on EUCAST breakpoints [60], because susceptible and resistant isolates are more useful than intermediate isolates for gaining initial mechanistic insights into nuc-aAST, and because susceptible and resistant are actionable calls in antibiotic-prescribing scenarios. Lastly, keeping in mind clinical utility, we timed each assay step to determine whether the nuc-aAST could yield a definitive susceptibility call within the time period of a patient’s visit, which is usually less than an hour [49,50].

## Results

### Design and rationale of the nuc-aAST

**Fig 1:**
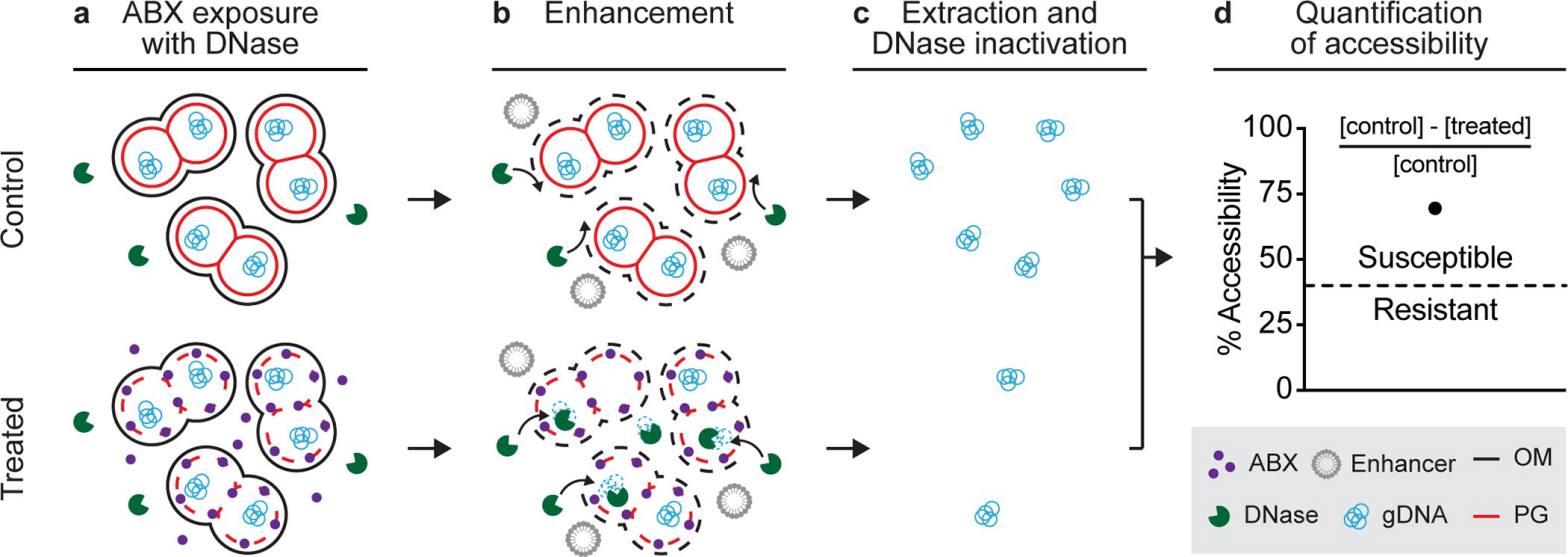
The nuc-aAST workflow shown for a sample containing a β-lactam susceptible pathogen. (**a**) A sample is split into control and treated aliquots; the treated aliquot is exposed to ABXs in the presence of DNase, and any extracellular DNA is digested. ABXs compromise PG of cells in the treated aliquot. (**b**) Accessibility to nucleases is enhanced by the addition of an enhancer, which disrupts the OM. gDNA becomes accessible and is degraded in the treated aliquot. Intact PG in control samples (or in treated but resistant samples) prevents degradation. (**c**) NAs are extracted, and DNase is inactivated. (**d**) Accessibility is quantified by measuring NA concentrations in the control and treated aliquots and dividing the amount of digested DNA by the amount in the control (to yield percentage accessibility). When the percentage accessibility is greater than the threshold (dashed line), the sample is categorized as susceptible. ABX, antibiotic; gDNA, genomic DNA; NA, nucleic acid; nuc-aAST, nuclease-accessibility antibiotic susceptibility testing; OM, outer membrane; PG, peptidoglycan.

The nuc-aAST method measures differences in the accessibility of genomic DNA to an exogenous nuclease between control and treated samples following a short antibiotic exposure. Like other NA-based AST methods, the nuc-aAST (Fig 1) relies on measuring changes in the quantity of pathogen-specific NAs in response to a treatment with an antibiotic; however, the nuc-aAST differs from existing NA-based ASTs in three aspects. First, in nuc-aAST, exposure of cells to β-lactams is performed in the presence of a DNase enzyme to degrade any DNase-accessible NAs (Fig 1A). DNA is accessible to DNase if it is released from the cells upon cell lysis or if the action of the antibiotic porates the cells and allows DNase to access the intracellular DNA. Second, in nuc-aAST, an enhancement step is introduced to increase accessibility of DNA in cells that have damaged or compromised peptidoglycan caused by β-lactams; DNase is present and active during this enhancement step (Fig 1B). Third, in nuc-aAST, lysis of the sample is performed only after DNase has degraded all accessible DNA (Fig 1C). This lysis step also inactivates the DNase, so that the enzyme does not impact downstream quantification (S1 Fig). Following inactivation of DNase and lysis, DNA remaining in the sample is quantified and the percentage of accessible DNA is used to determine susceptibility (Fig 1D). The percentage of accessible DNA is quantified by subtracting the concentration of inaccessible DNA (DNA not digested) in the treated aliquot from the concentration of DNA in the control aliquot and dividing this value by the concentration of DNA in the control. Measuring the percentage of accessible DNA is an NA-based metric that enables quantification of the damage to the cellular envelope induced by antibiotics targeting cell wall biosynthesis.

**Fig 2:**
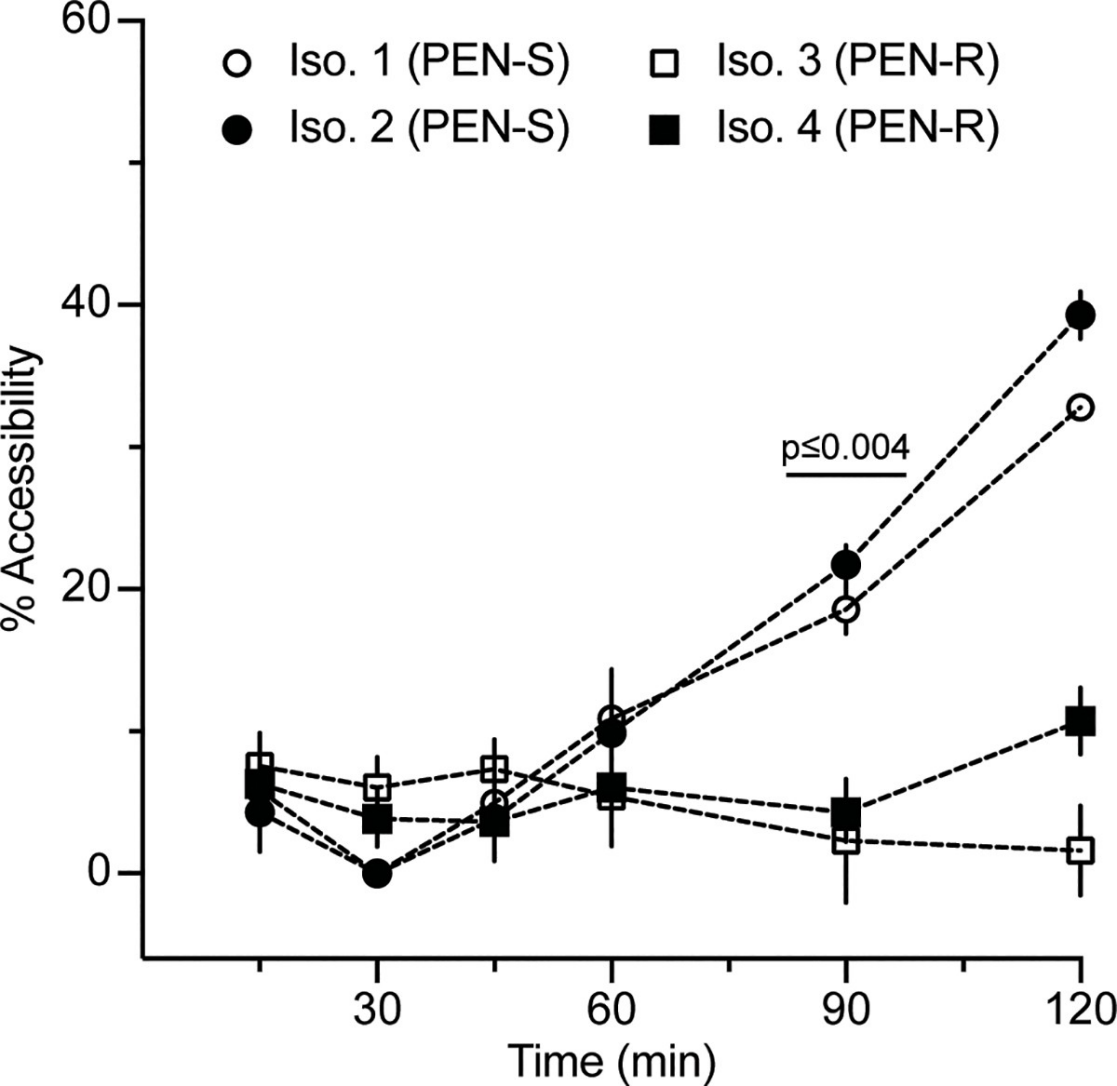
Percentage accessibility of DNA over time using the nuc-aAST without the addition of an enhancing step. Two PEN-S and two PEN-R *Ng* isolates were exposed to PEN in the presence of DNase I. DNA from the control and PEN-treated aliquots was extracted and quantified using qPCR at multiple time points to calculate percentage accessibility. Error bars represent the standard deviation of the PCR triplicates. Data are in S10 Table. *Ng*, *N*. *gonorrhoeae*; nuc-aAST, nuclease-accessibility antibiotic susceptibility test; PEN, penicillin; PEN-R, PEN-resistant; PEN-S, PEN-susceptible; qPCR, quantitative PCR.

β-lactams should primarily affect peptidoglycan [61] and should not have a major impact on the outer membrane (OM), which serves as a structural element in gram-negative bacteria [62]. Therefore, we expected the primary mechanism behind any increase in accessibility to be cell lysis as a result of exposure to β-lactams, leading to release of genomic DNA to the extracellular environment containing DNase. Additionally, we hypothesized that autolysis, which has been observed as an active stress response in *Ng* [63,64], might accelerate changes in accessibility due to antibiotic exposure. We tested our hypotheses in a time-course experiment using two PEN-susceptible and two PEN-resistant *Ng* clinical isolates (Fig 2). We observed a significant difference in the percentage accessibility between susceptible and resistant isolates after 90 min of exposure. This is the shortest incubation time for an *Ng* AST with PEN to date and faster than existing NA-based methods that rely on DNA replication [57]. However, the ideal length of an exposure step for an AST at the POC would be even shorter (15–30 min) to keep the entire workflow within the time period of a patient visit. Thus, we were compelled to further accelerate changes in accessibility of DNA to nuclease as a result of β-lactam exposure in susceptible samples.

### Enhancing changes in accessibility

We next hypothesized that the differences in DNA accessibility that we observed between susceptible and resistant isolates exposed to β-lactams could be enhanced using conditions that would increase the permeability of the cell envelope. In gram-negative organisms like *Ng*, the OM presents the first, and major, permeability barrier to macromolecules (e.g., nucleases and other enzymes) entering or exiting the cell, typically allowing only small molecules with molecular weights approximately <600 Da to pass through [65,66]. The peptidoglycan, in contrast to the OM, is a looser barrier that has been estimated to allow macromolecules up to 50 kDa to pass through [67–69]. We thus suspected that if the OM could be compromised, damage to the peptidoglycan would result in immediate, measurable changes in accessibility of genomic DNA to DNase, both by allowing DNase to enter and by allowing DNA fragments to exit. Therefore, we hypothesized that we could compromise the OM using an “enhancer” to decrease total assay time.

The ideal enhancer would (1) increase DNA accessibility to DNase in cells that have a compromised cell wall as a result of antibiotic exposure, (2) result in minimal lysis of healthy cells, (3) have a consistent effect on all *Ng* isolates, and (4) have no effect on downstream extraction and quantification of NAs. With these parameters in mind, we chose to test hypo-osmotic stress, stimulated autolysis, and four classes of surfactants as potential enhancers.

Hypo-osmotic stress was chosen as a method to enhance lysis of cells with damaged or compromised cell walls because osmotic stress of varying degrees is known to increase release of intracellular contents in gram-negative bacteria [70–72], although it has never been used to enhance accessibility in the context of AST. We exposed cells to hypo-osmotic conditions by diluting control and treated aliquots 20-fold in water with DNase I and 500 μM CaCl_2_, resulting in an approximately 244-mOsm/kg shift from the antibiotic-exposure conditions. Autolysis was chosen as an enhancer with the rationale of leveraging an already existing stress response in *Ng* to enhance changes in DNA accessibility. Autolysis is a natural stress response in *Ng* and can be accelerated by incubation in high-pH conditions (e.g., Tris [pH 8.5]) [73,74]. We hypothesized that using autolysis as an enhancer might result in large changes in NA accessibility. Surfactants were chosen as potential enhancers as a targeted chemical means of disrupting the bacterial cell membrane. We chose a representative surfactant from each of the four major charge-based classes of surfactants to investigate whether surfactant charge might lead to variability in their effectiveness due to natural variations in the OM of *Ng*. We tested the anionic surfactant sodium dodecyl sulfate (SDS), the cationic surfactant benzalkonium chloride (BAC), the nonionic surfactant TERGITOL NP (TNP), and the zwitterionic surfactant 3-[(3-Cholamidopropyl)dimethylammonio]-1-propanesulfonate (CHAPS). Each of these surfactant classes, with the exception of zwitterionic surfactants, have been well studied for their ability to compromise the integrity of the cell envelope [75], but none have been used in the context of AST or to change DNA accessibility on such short timescales. We chose to include the less well-studied zwitterionic surfactant CHAPS based on the diverse interactions of zwitterionic solutes with the bacterial cell envelope [76].

We tested each potential enhancer with respect to (1) the degree of lysis caused by incubation with the enhancer alone, (2) the ability to differentiate PEN-susceptible and PEN-resistant isolates using an enhancement step after exposure to PEN, and (3) the ability to differentiate CRO-susceptible and CRO-resistant isolates using an enhancement step after exposure to CRO, and (4) the ability to differentiate CFM-susceptible and CFM-resistant isolates using an enhancement step after exposure to CFM. We chose to use PEN, CRO, and CFM because we expected that the degree of change in NA accessibility as a result of enhancement would depend on the type of β-lactam used during exposure. CRO, CFM, and PEN bind and inhibit a different profile of penicillin-binding proteins [45,77] and have different rates of killing [78], which we expected would lead to different effects depending on the enhancer. Each enhancer was tested using multiple isolates susceptible or resistant to either PEN, CRO, or CFM. All enhancers were tested using a 5-min enhancement step after 15 min of antibiotic exposure. Antibiotic-exposure and enhancement steps were performed separately to decouple their effects on the *Ng* isolates.

**Fig 3:**
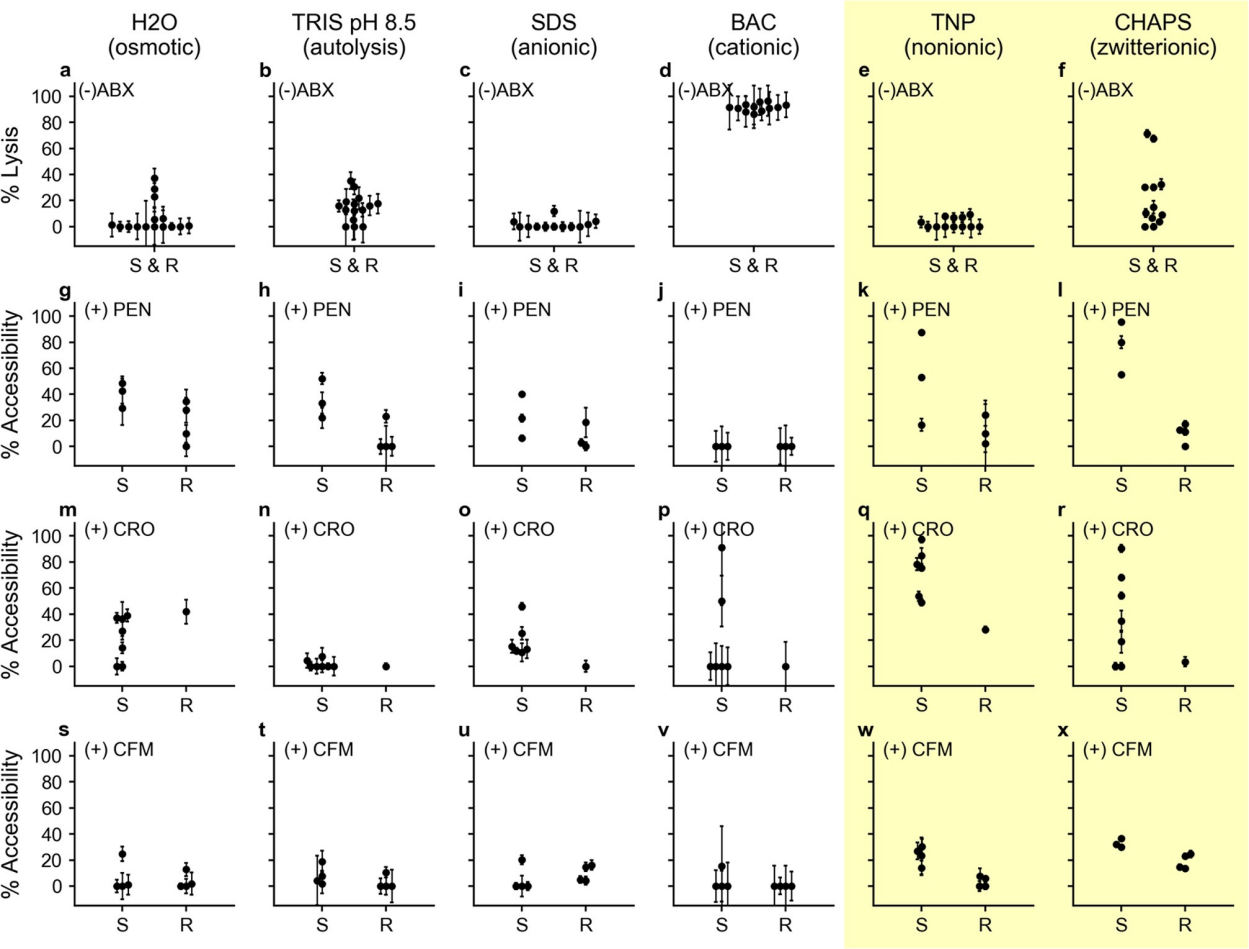
Selection of enhancers. (**a**-**f**) Six enhancers were tested for percentage of cell lysis due to enhancer alone (prior to ABX exposure). (**g**-**l**) Enhancement after 15-min exposure to PEN. (**m**-**r**) Enhancement after 15-min exposure to CRO. (**s-x**) Enhancement after 15-min exposure to CFM. Each point represents a clinical isolate run as a single experiment for that condition. All PCR was performed in technical triplicates with error bars representing the error in the PCR measurement propagated for the calculation of percentage lysis or percentage accessibility; all numerical values are available in S2 Table and S3 Table. The yellow shading indicates the two enhancers (TNP and CHAPS) most promising for nuc-aAST. Data for (a-f) are in S2 Table and (g-x) are in S3 Table. ABX, antibiotic; BAC, benzalkonium chloride; CFM, cefixime; CHAPS, 3-[(3-Cholamidopropyl)dimethylammonio]-1-propanesulfonate; CRO, ceftriaxone; nuc-aAST, nuclease-accessibility antibiotic susceptibility testing; PEN, penicillin; R, resistant; S, susceptible; SDS, sodium dodecyl sulfate; TNP, TERGITOL NP.

Enhancers were first tested for the degree of lysis caused by a 5-min incubation with the enhancer alone (Fig 3A–3F). If the enhancement step lyses the majority of cells even without antibiotic exposure, then DNA accessibility will increase in both control and treated aliquots, and any effect of the antibiotic will be diminished. We observed an average of <50% lysis when testing all potential enhancers except BAC (Fig 3D), which showed an average of 91.6% lysis across all 12 isolates tested (See S2 and S3 Tables for data).

We next measured the percentage DNA accessible when using each enhancer after a 15-min exposure to PEN. We evaluated the ability to differentiate PEN-susceptible and PEN-resistant isolates based on the average percentage accessibility in susceptible isolates (which we want to be large), the average percentage accessibility in resistant isolates (which we want to be small), and the magnitude of separation between those two values. Based on these criteria, Tris (Fig 3H), TNP (Fig 3K), and CHAPS (Fig 3L) were the most promising enhancers for differentiating PEN-susceptible and PEN-resistant isolates after 15 min of exposure. However, we observed differences in DNA accessibility in response to CRO and CFM compared with PEN depending on the enhancer used (Fig 3M–3X). Among CFM-susceptible isolates, the responses to antibiotics and each enhancer were smaller than in PEN-susceptible and CRO-susceptible isolates. TNP and CHAPS were the only tested enhancers that enabled us to differentiate CRO-susceptible and CRO-resistant responses (Fig 3W–3X) after 15 min of CFM exposure. We were unable to observe consistently large changes in the seven tested CRO-susceptible isolates using the other two ionic surfactants, SDS (Fig 3I and 3O) and BAC (Fig 3J and 3P), regardless of the antibiotic treatment. Following these tests, we chose CHAPS as the enhancer to use for validation of the nuc-aAST with clinical isolates because it resulted in low-percentage lysis, large increases in DNA accessibility for PEN-susceptible and CRO-susceptible isolates following exposure, and only small increases in the DNA accessibility of PEN-resistant, CRO-resistant, and CFM-resistant isolates.

### Validation using clinical isolates

**Fig 4:**
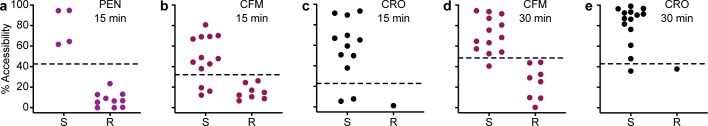
Validation of nuc-aAST using clinical isolates. (**a-c**) nuc-aAST results after 15 min of exposure to (**a**) PEN, (**b**) CFM, and (**c**) CRO. (**d-e**) nuc-aAST results after exposure to (**d**) CFM and (**e**) CRO for 30 min. Each point represents the average for a single isolate run in (at least) biological triplicate for that condition. All PCR was performed in technical triplicate. The dashed line represents the susceptibility threshold, which was set at 26.5% accessibility for 15-min exposures and 46% for 30-min exposures. Data plotted are in S4 Table; experimental data from individual replicates are detailed in S11 Table and S12 Table. AST, antibiotic susceptibility testing; CFM, cefixime; CRO, ceftriaxone; nuc-aAST, nuclease-accessibility AST; PEN, penicillin; R, resistant; S, susceptible.

To validate the nuc-aAST, we performed 48 ASTs (with at least three biological replicates each) using 21 clinical isolates of *Ng* exposed individually to PEN, CFM, or CRO for 15 min. We then compared the categorical susceptibility determined using the nuc-aAST to the susceptibility determined using gold-standard agar dilution (Fig 4A–4C). Receiver operating characteristic (ROC) plots [79] (S4 Fig) were created so that the area under the curve (AUC) could be calculated separately for each β-lactam tested. After 15 min of exposure, we obtained an AUC of 1.000 (PEN), 0.875 (CFM), and 1.000 (CRO). The AUC is determined by scanning a threshold through the ROC plot and measuring the sensitivity and specificity at each theoretical threshold value. This scanning allows one to select the threshold that would differentiate susceptible and resistant organisms with the maximum sensitivity and specificity within the given dataset. For example, an AUC of 1.000 indicates there was a threshold value that perfectly separated susceptible and resistant categories. However, AUC measurements do not consider the experimental noise or the magnitude of separation between susceptible and resistant samples and should be applied with care to datasets with limited numbers of measurements, such as ours. For example, in the case of CRO, the difference between the single CRO-resistant isolate that was available to us and the two CRO-susceptible isolates with the lowest responses was small after 15 min of exposure. Therefore, setting the susceptibility threshold between them would be impractical, even though it would yield 100% categorical agreement. We therefore decided to set a single threshold for all three antibiotics at a more conservative 26.5% even though this threshold generates some errors in both the CFM and CRO measurements after 15 min of antibiotic exposure. This threshold was chosen because it generated the fewest number of errors with 15 min of CFM exposure. The exact value is the average of the closest susceptible and resistant isolate.

We then hypothesized that the differences observed in the magnitude of the response of the susceptible isolates after 15 min of exposure to each antibiotic, including the errors observed when testing CFM and CRO, could be the result of differences in how fast each β-lactam affects *Ng* [78]. For example, a possible explanation for differences among isolates in their response to antibiotics could be phylogenetic differences [80–82]. If isolates differ in their response times, a longer exposure would result in larger average separation between susceptible and resistant isolates and potentially better categorical agreement if the susceptible isolates were less responsive as a result of a delayed response to antibiotic.

To test the hypothesis that there are inherent differences in isolate response time, we performed nuc-aAST using CFM and CRO with 30-min exposure times, and as predicted, we observed a larger average separation between susceptible and resistant isolates and only a single error with each antibiotic. After 15 min of exposure to CFM and CRO, 77% and 83% of susceptible isolates, respectively, were classified as susceptible using nuc-aAST. After 30 min of exposure to CFM and CRO, 95% and 92% categorical agreement was obtained for CFM and CRO, respectively. The AUC for CFM and CRO after 30 min of exposure were 0.917 and 0.981, respectively (S4 Fig).

### Pilot nuc-aAST tested directly on clinical urine samples

Our long-term goal, well beyond the scope of this manuscript, is to develop phenotypic AST assays and devices for clinical settings. As a proof of concept that our nuc-aAST method is aligned with that goal, we performed pilot nuc-aAST experiments directly on fresh clinical urine samples without a culturing step (see Methods). We note that a phenotypic *Ng* AST has never been successfully performed directly on clinical samples by any method; the gold-standard AST requires isolation of the pathogen, and then the AST is performed on the isolate. At the time we began this pilot, it was unknown whether it would even be possible to obtain an AST result directly from a clinical sample without a culturing step. The stability and viability of *Ng* in urine samples has never been characterized, and to reduce the unknown variables, these proof-of-concept nuc-aAST were performed on fresh clinical samples. We set up a satellite lab at the AIDS Healthcare Foundation (AHF) clinic where samples were obtained. The nuc-aAST method depends on DNase I functionality, so we first ran control experiments with a spike-in of lambda DNA into three different urine samples to show that DNase I remained active under the conditions and timeframe we are testing (data shown S2 Fig). All clinical urine samples reported herein were run immediately after collection (in all four samples, the sample handling began within 30 min of sample donation). Not all clinical samples were positive or yielded AST results (some samples were negative for *Ng* and some could not be reliably quantified; see Methods for details). Six nuc-aASTs were obtained directly on four clinical urine samples, including four with PEN and two with CRO. All nuc-aAST experiments were performed with technical triplicates.

**Fig 5:**
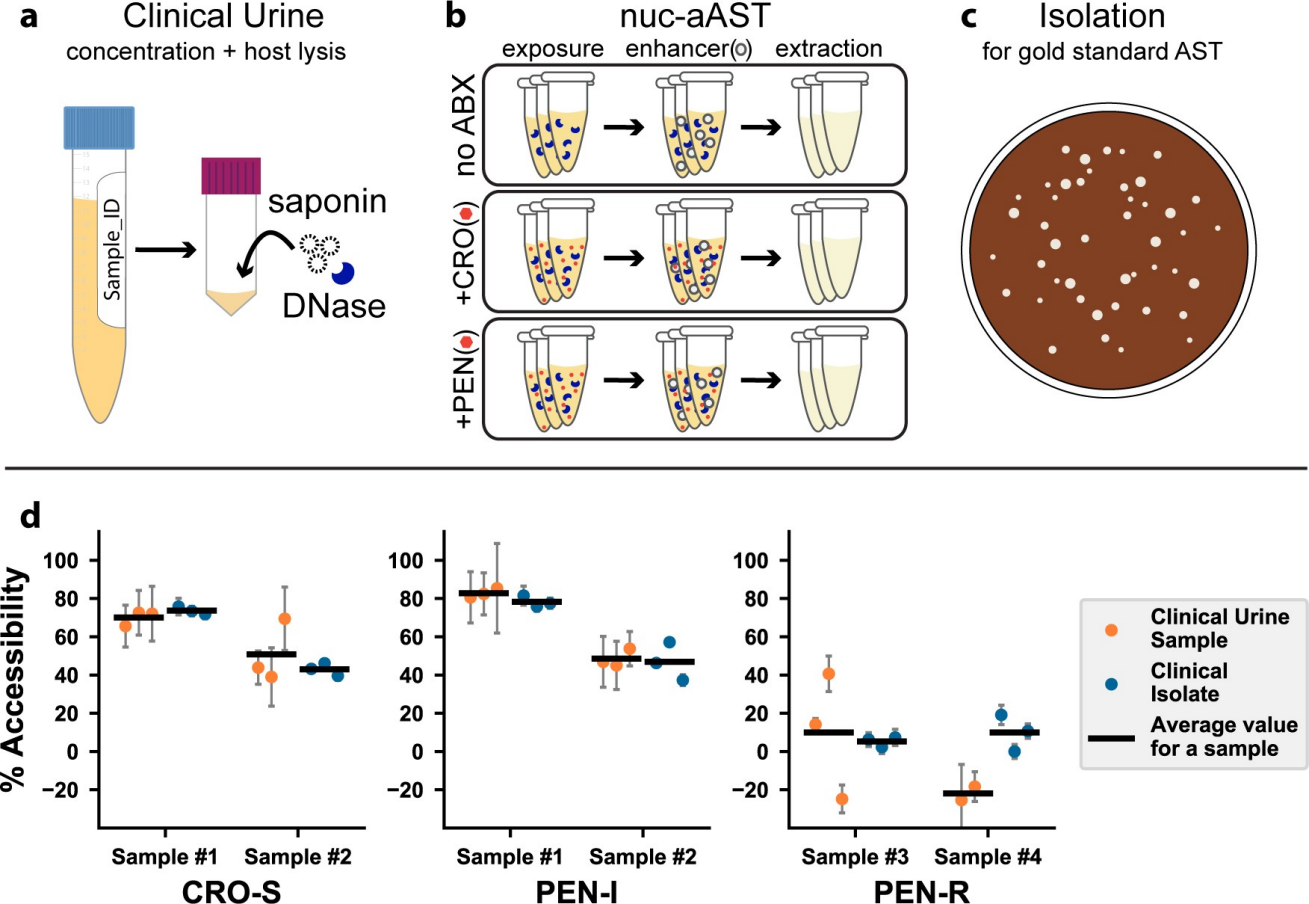
Pilot nuc-aAST with clinical urine samples. (**a**) Clinical urine samples were concentrated by a factor of 5 and incubated with DNase I and saponin for 15 min to lyse host cells and clear free DNA. (**b**) The nuc-aAST protocol was performed in technical triplicates with no ABX, CRO, or PEN. Each nuc-aAST consisted of a 30-min exposure to 1 μg/mL CRO or PEN followed by a 3–5 min exposure to the enhancer TNP (see Methods for details). (**c**) Each clinical sample was isolated for gold-standard MIC testing and further experiments. (**d**) Results of six nuc-aASTs were performed directly on four clinical urine samples and susceptibility was determined (orange points). In parallel, isolates were prepared from these clinical samples, and the results of the rapid nuc-aAST with clinical urine samples were compared to the same protocol with the prepared isolate (blue points). The means of the replicates are shown as horizontal lines (S7 Table). The MIC was determined by the gold-standard method (S1 Table). Error bars are the error in the PCR measurement propagated for the calculation of percentage lysis or percentage accessibility (S6 Table). ABX, antibiotic; AST, antibiotic susceptibility testing; CFM, cefixime; CRO, ceftriaxone; CRO-S, CRO-susceptible; MIC, minimum inhibitory concentration; nuc-aAST, nuclease-accessibility AST; PEN, penicillin; PEN-I, PEN-intermediate; PEN-R, penicillin-resistant; TNP, TERGITOL NP.

To perform nuc-aAST and gold-standard comparison, each clinical urine sample was divided into two parts; one part was used immediately to run the nuc-aAST and one part was cultured to obtain the isolates for the gold-standard culture-based AST. For the nuc-aAST (Fig 5A and 5B), clinical urine was first centrifuged to concentrate, then resuspended in culture media with saponin (to selectively lyse host cells) and DNase I (to clear free DNA from host and dead bacterial cells) for a 15-min incubation [83–85]. The suspension was centrifuged again and then resuspended in fresh Graver-Wade medium (GWM). Next, the sample was exposed to antibiotics (PEN or CRO) or the control solution (nuclease-free water [NF-H_2_O]) for 30 min. Then, all samples were exposed to the enhancer TNP for 3–5 min. DNA concentrations were measured with quantitative PCR (qPCR) and the data analyzed as described in the Methods. The results from each experimental replicate are shown in Fig 5D.

To perform the gold-standard culture-based AST, the second part of each of the four clinical urine samples was plated and subcultured on selective media, and over the course of 3–5 d, isolates were prepared (details in “MIC testing and creation of clinical isolates” in the Methods). Gold-standard agar-dilution minimum inhibitory concentration (MIC) testing was performed on these isolates, and the resulting MICs are reported in Fig 5D and S1 Table. We emphasize that gold-standard AST information was obtained days after nuc-aAST experiments. The isolates from each of these four clinical urine samples were then handled identically to the urine samples, including saponin and DNase I pretreatments, and the nuc-aAST was repeated. The results of the direct-from-sample nuc-aAST and the results of the nuc-aAST on the isolate from these clinical samples are reported in Fig 5D to compare the performance. The nuc-aAST results of the clinical urine sample and the isolate carried out with the same method gave comparable results; notably, the clinical data are (as expected) more variable among replicates, which is partially the result of bacterial loads in the urine sample being lower than in the assay run on isolates. A threshold at 46% accessibility (Fig 4) correctly categorized all six ASTs from the clinical samples (S7 Table) as antibiotic resistant or not antibiotic resistant.

The pilot nuc-aAST experiments with clinical samples were run with a slightly modified workflow compared with the isolates. For example, we used a slightly shorter enhancement step, and we used TNP instead of CHAPS as the enhancer to minimize background lysis of bacterial cells. Additionally, we only encountered PEN-intermediate (PEN-I) *Ng* and PEN-resistant *Ng*. Because we are testing one concentration of antibiotics (1 μg/mL) in the nuc-aAST, which is above the MIC of the PEN-I *Ng* and below MIC of the PEN-resistant *Ng*, as expected PEN-I *Ng* gave a susceptible (or “not resistant” [NR]) call and PEN-resistant *Ng* gave a resistant call.

### Sum-of-steps total time using contrived urine samples

To make a more realistic measure of total assay time, we modified the extraction and quantification steps of the nuc-aAST. The exposure and enhancement steps were performed as described previously, but NA quantification was performed using a rapid, chip-based, digital loop-mediated isothermal amplification (dLAMP) method, as described previously [86]. Additionally, we used a faster, single-step NA extraction method based on previous work [51]. Both modifications made the workflow faster. Additionally, the high precision of digital quantification allowed us to make a susceptibility call as soon as there was a significant difference between the concentration of NAs in the control and treated aliquots.

**Fig 6:**
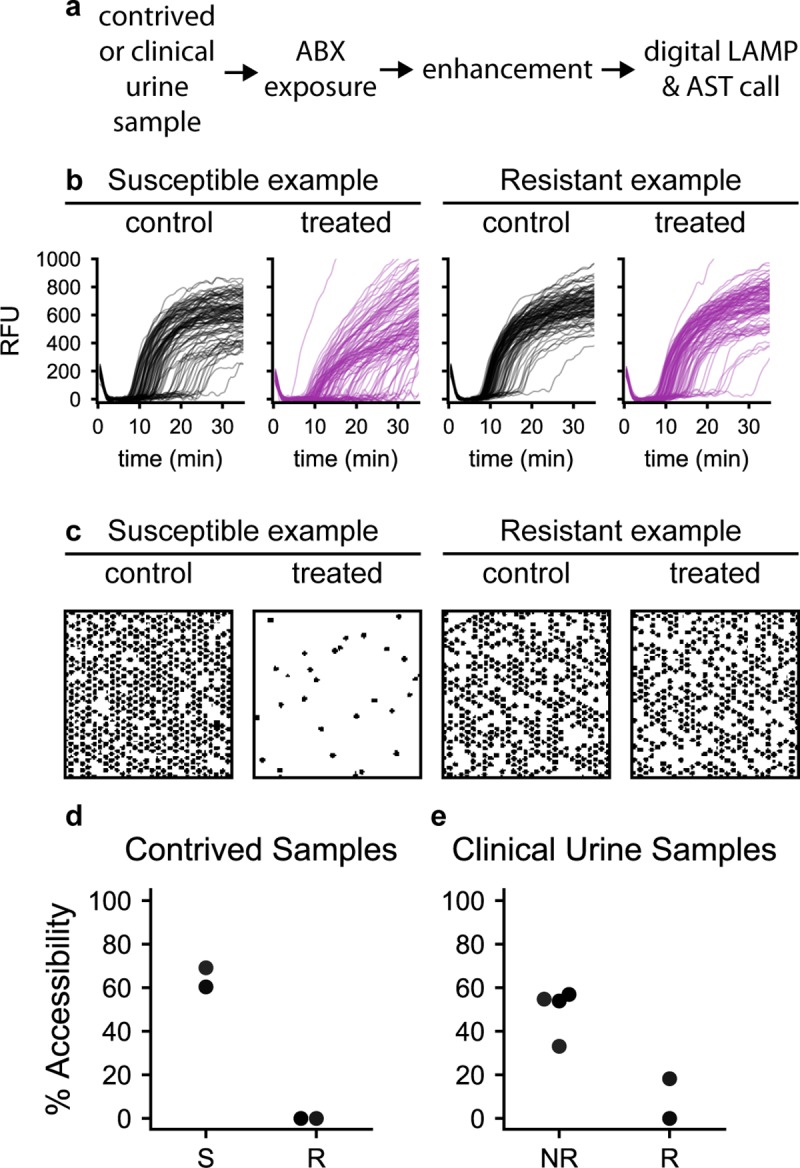
The nuc-aAST workflow for contrived and clinical urine samples with each step timed. (**a**) The nuc-aAST workflow times required for contrived samples are 15-min ABX exposure, 5-min enhancement, 2 min for DNA extraction, 8 min for digital LAMP and AST call; timing for clinical urine samples includes 30-min ABX exposure, 3–5-min enhancement, 10 min for extraction, and 20 min for digital LAMP and AST call. (**b**) Amplification curves from which positive wells were determined and counted (a subset of 100 wells is shown for each microfluidic chip, S1–S4 Data) (**c**) A 2 × 2-mm subsection of masks was created from chips used for performing digital LAMP on control and antibiotic-treated aliquots of S and R samples; as an illustration, each mask shows approximately 625 wells (out of about 20,000 total wells) after 10 min of amplification. Wells that showed amplification of *Ng* DNA appear black. (**d**) The percentage of accessible DNA was determined at earliest significance (7–8 min of amplification; see Methods and S5 Table) for two penicillin-S and two penicillin-R samples run using digital LAMP (S5 Table). Each step was timed individually, and the sum of steps of the assay was 30 min. (e) Percentage accessibility was determined after 20 min of digital LAMP for a representative technical replicate for each of the six nuc-aAST performed directly on four clinical urine samples. Ceftriaxone-S and penicillin-intermediate samples are plotted together as NR (S5 Table; MICs are in S1 Table). ABX, antibiotic; AST, antibiotic susceptibility testing; LAMP, loop-mediated isothermal amplification; MIC, minimum inhibitory concentration; *Ng*, *N*. *gonorrhoeae*; NR, not resistant; nuc-aAST, nuclease-accessibility AST; R, resistant; RFU, relative fluorescence units; S, susceptible.

We measured total assay time based on the sum of the steps of the nuc-aAST using contrived urine samples. Contrived samples mimic clinical urine samples and allowed us to better evaluate how the assay would perform in a clinical setting compared with assays performed with isolates in media. Samples were created using two PEN-susceptible and two PEN-resistant isolates; one of the two PEN-resistant isolates was positive for β-lactamase activity, which we included in order to have PEN-resistant isolates with different mechanisms of resistance. To perform the AST, samples were first split into control and treated aliquots and incubated at 37°C for 15 min. Next, the samples were transferred to the enhancement step and incubated for 5 min in the presence of CHAPS. Samples were then extracted as described previously, and dLAMP was performed in commercial chips [86]. Images were obtained in real time using a custom imaging system [87]. LAMP quantification was performed using an automated data-analysis workflow in MATLAB [86] in which images are automatically processed and positive wells counted based on a digitized mask created from the final image (Fig 6B). NA concentrations were used to determine percentage accessibility as soon as the measured NA concentrations in the susceptible sample became significantly different between the control and treated chips. All samples were tested in a total time (measured as the sum of steps) of 30 min and agreed with gold-standard agar dilution (Fig 6D). Performance of dLAMP was evaluated on extractions from clinical sample nuc-aAST experiments (Fig 6E). The extension of the dLAMP reaction is a result of low NA concentrations present in the clinical samples (data shown in S5 Table).

## Discussion

Here, we described a new approach—nuc-aAST—to enable developing a critically needed rapid phenotypic AST for the globally important pathogen *Ng*. We show that by measuring the change in the accessibility of DNA after 15- or 30-min β-lactam exposure, the nuc-aAST yields a phenotypic susceptibility readout in less than 1 h, as opposed to the currently available methods, which require hours to days. We further show that the nuc-aAST breaks the current speed limits for NA-based phenotypic ASTs using β-lactams (which do not directly impact NAs) by using an innovative approach: coupling cell wall damage to NA readout. The nuc-aAST thus provides a new approach for designing rapid phenotypic ASTs with NA-based readouts for antibiotics that impact cell envelope integrity. Overall, we envision that leveraging the nuc-aAST and combining it with other creative biological and chemical insights will result in similarly innovative approaches for other important antibiotic classes for *Ng*, such as protein-biosynthesis inhibitors like TET and AZM. Existing NA-based approaches, such as those for CIP [25,56], can also be combined alongside the nuc-aAST.

We found that phenotypic ASTs that use NA accessibility as a readout benefit from the use of a carefully chosen enhancer. Here, the enhancement step consisted of a surfactant (CHAPS) that enabled detection of cell wall damage faster than cell division. Without the enhancement step, the cell envelope remains intact longer, so measurements of DNA accessibility approximate the timescale of cell division (Fig 2), which, for fastidious organisms such as *Ng*, will be too slow for POC applications. Furthermore, on the timescales tested here, DNA release in susceptible, treated samples may in part be the result of β-lactams “porating” the cell wall or cells lysing as a result of stress. Cell wall turnover may also proceed even if cells are not actively dividing. In both cases, the enhancement step is critical to shorten the time required to detect a difference between control and antibiotic-treated samples. The increase in DNA accessibility in susceptible isolates will differ based on the combination of β-lactam used and enhancer, highlighting the importance of testing multiple β-lactams with the nuc-aAST. Of the surfactants tested as enhancers, the charge-neutral surfactants TNP and CHAPS gave better results than the ionic surfactants SDS and BAC, suggesting that charge may be an important factor when designing an effective enhancement step. We also anticipate that organism-specific OM chemistry and general stress responses will play a role in determining which enhancers are optimal in other organisms.

We found that for PEN, susceptibility of *Ng* could be determined after just 15 min of exposure in all isolates tested (Fig 4A). However, for CFM and CRO, a small number of susceptible isolates did not respond after 15 min of exposure, but almost perfect categorical agreement was obtained after 30 min of exposure (Fig 4B–4E). We hypothesize that CFM and CRO required a longer exposure than PEN because of their differences in binding kinetics and rates of killing [77,78,88]. Despite these differences, an actionable susceptibility call (i.e., determining that a susceptible isolate is susceptible to a particular antibiotic and therefore can be treated with that antibiotic) could still be made for most isolates after 15 min of exposure. The errors yielded from the nuc-aAST at 15 min of antibiotic exposure would not result in ineffective treatment because these errors are from susceptible isolates (three CFM-susceptible and two CRO-susceptible) that were misidentified as resistant. We emphasize that in our isolate dataset, these errors are reduced to one CFM-susceptible and one CRO-susceptible isolate by extending the antibiotic incubation time by just 15 min. One approach to balance reducing assay time with minimizing errors is to perform two exposures in parallel for each antibiotic. The first exposure would be analyzed after 15 min. If a response is obtained indicating that the pathogen is susceptible (which should be the case for the majority of patients), the second exposure would be discarded. If no response or if an equivocal response is obtained, then the second exposure (after 30 min total) would be analyzed to provide the definitive susceptibility call. With this approach, the test would provide the answer after 15 min of antibiotic exposure for the majority of patients, and only a few patients would be delayed by the additional 15 min of antibiotic exposure.

Several limitations will need to be overcome in order to translate the nuc-aAST approach to an automated and distributable system. First, in this paper, we used clinical isolates, contrived urine samples, and four clinical urine samples. Although contrived samples are a good proxy for clinical samples and are accepted by the US Food and Drug Administration in certain cases [89], we also wished to perform a pilot experiment to demonstrate the nuc-aAST can work directly on fresh clinical samples. Our six nuc-aAST experiments gave good agreement with the ASTs that were run on isolates taken from the same samples (Figs 5 and 6). These pilot data suggest that the nuc-aAST can be adapted to work directly on clinical urine samples without the need for a culturing step and can utilize existing dLAMP techniques from crude lysate of a clinical sample. Because there is no rapid (20 min) POC *Ng* ID test currently available, we had to run ASTs without knowing whether the samples are positive for *Ng*; as expected, not all clinical samples collected yielded interpretable AST data (see Methods). Further development will be needed to optimize the nuc-aAST for clinical use. Performing phenotypic AST on clinical samples is extremely challenging and has only been demonstrated a few times, many from clinical urinary tract infection (UTI) samples [51,53,90,91]. This manuscript is a demonstration of a phenotypic AST used directly on *Ng* clinical samples. Many recent breakthroughs in phenotypic AST are initially reported without any validation with clinical samples; most papers use isolates [56,81], contrived samples [57,92–94], or positive blood cultures [95] instead of raw clinical samples. Second, future work should test more *Ng* isolates to encompass broader phylogenetic diversity [80–82] when they are made available to researchers and characterized, as well as test isolates with intermediate resistance to PEN, CFM, and CRO. These efforts could also aim to establish a correlation in the magnitude of nuc-aAST response and MIC of antibiotics, which would provide even more detailed information at the POC. Third, in timing the sum of steps, we did not include handling time; future work should include optimization of handling steps and timed sample-to-answer experiments. Finally, the nuc-aAST method will need to be translated to a POC device so that larger-scale clinical evaluations can be performed. Devices for multiplexed digital quantification [96–98] have been demonstrated and would be useful in performing nuc-aAST for multiple antibiotics in parallel.

We envision that nuc-aAST would be deployed in combination with two complementary technologies: (1) the pathogen ID technologies that are being developed by others [20,21,23,24] to identify *Ng*-positive samples that require an AST and (2) rapid genotypic and/or phenotypic ASTs that rely on NA readouts for other antibiotics used in the treatment of *Ng*, including fluoroquinolones (CIP) [25,51,81] and protein synthesis inhibitors (TET and AZM) [81]. Assuming these two complementary technologies are developed and validated, further development of nuc-aAST would provide the last—and we would argue the most challenging—piece needed for a complete rapid ID/AST workflow for *Ng* based on NA readout. We chose NA readout for the nuc-aAST for two reasons. First, NA readouts will enable easy integration with pathogen ID and other NA-based AST technologies. Second, NA readouts are organism specific [51] and therefore should be effective even for mixed microbial populations potentially present in clinical samples (e.g., *Ng* in the presence of commensals or other pathogens).

Implementation of a rapid phenotypic AST would dramatically improve the antibiotic stewardship of *Ng* infections and therefore impact the health of people who are infected with *Ng*; currently, there are an estimated 78,000,000 global cases of *Ng* every year [99]. Furthermore, the nuc-aAST approach provides a framework for developing additional accessibility-based AST chemistries for other pathogens that pose global-health threats but have been challenging for current phenotypic AST methods. For example, we have shown that quantifying NA accessibility to polymerases can be used to rapidly determine antibiotic susceptibility in Enterobacteriaceae [100]. Overall, this work highlights the diagnostic capabilities that can be attained by developing innovative NA-based assays for AST; further expansion and application of these approaches are critically needed to address the crisis posed by antibiotic-resistant bacteria.

## Methods

### Ethics statement

Clinical urine samples were collected at the AHF clinic under Caltech IRB #18–0865 from consented male patients symptomatic for *Ng*.

### Study design

The objective of this study was to develop a rapid phenotypic AST for β-lactams based on DNA accessibility to nuclease for use with *Ng*. The key hypotheses of this work were as follows: (1) following antibiotic exposure, DNA in susceptible cells would be more accessible to an exogenously added nuclease than DNA in resistant cells because of cell wall damage as a result of exposure to antibiotics; (2) this difference would occur faster than cell division; and (3) this difference in DNA accessibility could be detected sooner if a surfactant enhancement step was included. To test the first two hypotheses, we performed an exposure time course using two PEN-susceptible and two PEN-resistant clinical isolates of *Ng*. To test the third hypothesis, we performed 48 nuc-aASTs using 21 clinical *Ng* isolates (exposed to antibiotics for 15 min) and 36 nuc-aASTs using 21 clinical *Ng* isolates (exposed to antibiotics for 30 min). We then compared the results to gold-standard agar dilution.

To calculate the sample size, the methods and Eq 5 from [101] were used as described previously [51]. Namely, we suspected that the specificity and sensitivity of the nuc-aAST method would be 95% with a desired margin of error of ±10%. Under these conditions, 18.2 (or 19) samples must be tested with the nuc-aAST method and compared to the gold standard. For 15-min antibiotic exposures, we performed 29 ASTs with isolates susceptible to the antibiotic being tested and 19 ASTs with isolates resistant to the antibiotic being tested.

### Isolates and agar-dilution MIC testing

Isolates were provided by the University of Washington Neisseria Reference Laboratory and the CDC Antibiotic Resistance (AR) Isolate Bank *N*. *gonorrhoeae* panel (S1 Table). MICs of the CDC AR Isolate Bank are reported by the CDC [102], and MICs of all other isolates were determined by agar dilution according to the Clinical and Laboratory Standards Institute (CLSI) guidelines [103].

### Reagents and culture media

BD BBL Chocolate II Agar prepared plated media (GC II Agar, with Hemoglobin and BD IsoVitaleX) was purchased from VWR International LLC (VWR, Radnor, PA). GWM was prepared as described previously [38]. Cation-adjusted Mueller Hinton II Broth (MHB) (BD, Franklin Lakes, NJ) was prepared according to manufacturer instructions. All sodium bicarbonate (NaHCO_3_) (Sigma, St. Louis, MO) and calcium chloride (CaCl_2_) (Fisher Scientific, Hampton, NH) stocks were dissolved in NF-H_2_O and sterilized using 0.2-μm filters. DNase I (2,000 U/mL) was obtained from New England Biolabs (NEB; Ipswich, MA). Normal urine from pooled human donors was purchased from Lee Biosolutions (Maryland Heights, MO) and filtered through 0.2-μM filters before use.

Antibiotic stocks were prepared and stored as single-use aliquots at −80°C. Aliquots were thawed once and diluted in NF-H_2_O before use. PEN (1 mg/mL) was prepared from penicillin G sodium salt (Sigma, St. Louis, MO) in NF-H_2_O. CRO (1 mg/mL) was prepared from ceftriaxone disodium salt hemi(heptahydrate) (Sigma) in NF-H_2_O. CFM (5 mg/mL) was prepared from cefixime trihydrate (Sigma) in DMSO.

Unless otherwise noted, enhancer stock solutions were prepared in NF-H_2_O and stored at room temperature. Tris buffer (500 mM [pH 8.5] at 37°C) was prepared according to the Sigma buffer reference tables [104] using 0.2-μm filter sterilized stocks of 1 M Tris-HCl (Sigma) and 1 M Tris base (Fisher Scientific) prepared in milliQ H_2_O. TNP HLB 13.1 (100 mM) was prepared by mixing 334 μL 100 mM Tergitol NP-9 (Sigma) + 666 μL 100 mM Tergitol NP-10 (Sigma). CHAPS (200 mM) was prepared from CHAPS solid powder (Sigma). SDS (0.1%) was prepared by diluting 10% SDS (Invitrogen, Carlsbad, CA). BAC (10%) was prepared from benzalkonium chloride solid powder (MP Biomedicals, Santa Ana, CA).

### NA quantification

qPCR was performed using ssoFast EvaGreen Supermix (Bio-Rad Laboratories, Hercules, CA) in 10 μL reactions with 500 nM primers targeting the *Ng* 16S rRNA gene [105]. DNA template composed 10% of the reaction volume. Cycling conditions consisted of 3.0 min at 95°C, followed by 35 cycles of 15 sec at 95°C, 15 sec at 62°C, and 20 sec at 72°C. All qPCR was performed on either a Roche LightCycler 96 or Bio-Rad CFX96 instrument. The Cq values obtained from qPCR are used to compute the percentage accessibility and percentage lysis as described in the following equations. Any negative percentages were set to 0 for plotting.

$$\%Accessibility\;(control\;and\;treated)=(1-2^{\left({cq}_{control}-{cq}_{treated}\right)})\times 100$$ (1)

$$PCR\;error\;\%\;Accessibility=\sqrt{{\left(\frac{\sigma_{C}}{\mu_{C}}\right)}^{2}+{\left(\frac{\sigma_{T}}{\mu_{T}}\right)}^{2}}\times 100$$ (2)

- $C=linearized\;control\;Cqs=2^{Cq_{control}}$
- $T=linearized\;treated\;Cqs=2^{Cq_{treated}}$
- σ = standard deviation
- μ = mean

$$\%\;Lysis\;(no\;enhancer\;and\;enhancer)=(1-2^{\left({cq}_{no\;enhancer}-{cq}_{enhancer}\right)})\times 100$$ (3)

$$PCR\;error\;\%\;Lysis=\sqrt{{\left(\frac{\sigma_{N}}{\mu_{N}}\right)}^{2}+{\left(\frac{\sigma_{E}}{\mu_{E}}\right)}^{2}}\times 100$$ (4)

- $N=linearized\;no\;enhancer\;Cqs=2^{Cq_{no\;enhancer}}$
- $E=linearized\;enhancer\;Cqs=2^{Cq_{enhancer}}$
- σ = standard deviation
- μ = mean

Droplet digital PCR (ddPCR) was performed using QX200 ddPCR Supermix for EvaGreen (Bio-Rad) with the same primers and primer concentrations used in qPCR. DNA template composed 10% of the reaction volume. Cycling conditions consisted of 5.0 min at 95°C, followed by 40 cycles of 30 sec at 95°C, 30 sec at 60°C, and 30 sec at 72°C, followed by a droplet stabilization step of 4°C for 5 min and 95°C for 5 min. Calculations of percentage accessibility and percentage lysis for ddPCR are given in the following equations, where λ represents template concentration in copies/μL. The template concentrations are used to compute percentage accessibility and percentage lysis as described in the following equations. Any negative percentages were set to 0 for all analyses.

$$\%\;Accessibility\;(control\;and\;treated)=\left(1-(\frac{\lambda_{treated}}{\lambda_{control}})\right)\times 100$$ (5)

$$\%\;Lysis\;(no\;enhancer\;and\;enhancer)=\left(1-(\frac{\lambda_{enhancer}}{\lambda_{no\;enhancer}})\right)\times 100$$ (6)

A dLAMP assay was performed using a previously published system [86]. The dLAMP mix consisted of 1 μL NEB Isothermal Amplification Buffer (200 mM Tris-HCl, 20 mM MgSO_4_, 500 mM KCl, 100 mM [NH_4_]_2_SO_4_, 1% Tween 20 [pH 8.8]), 0.6 μL MgSO_4_, 0.5 μL BSA (20 mg/mL), 0.4 μL Syto-9 (50 μM, prepared within 2 wk of use), 1.4 μL dNTPs (10 mM each), 0.5 μL 20X primer mix, 0.4 μL NEB Bst 2.0 WarmStart, 0.2 μL Ambion RNase cocktail, 4.0 μL NF-H_2_O, and 1 μL of template. Primers were designed to target the *Ng* 16S gene and screened as described previously [51]. Primer sequences used are as follows, with the final concentration in the amplification mix in parentheses: GCGGTGGATGATGTGGATT (forward outer primer, 0.2 μM), CCGGCAGTCTCATTAGAGTG (backward outer primer, 0.2 μM), CTCCTCCGTCTCCGGAGGATTCaaaaCGATGCAACGCGAAGAAC (forward inner primer, 1.6 μM), TCGTCAGCTCGTGTCGTGAGATttttCCCAACCGAATGATGGCA (backward inner primer, 1.6 μM), CGCACATGTCAAAACCAGG (forward loop primer, 0.4 μM), and GCAACGAGCGCAACCC (reverse loop primer, 0.4 μM). Eq 3 was used to compute percentage accessibility, where λ represents the template NA concentration in copies/μL as measured by dLAMP.

### *Ng* culture preparation

Isolates were streaked from glycerol stocks stored at −80°C onto BD BBL Chocolate II Agar plates and incubated overnight in a 37°C incubator with 5% CO_2_. Isolates were then passed onto fresh BD BBL Chocolate II Agar plates and grown for 4–7 h at 37°C with 5% CO_2_. In all experiments, cells from plates passed 1–3 times were used. Several colonies were scraped and resuspended in 37°C GWM to generate a working suspension. Optical density at 600 nm (OD_600_) was measured, and the working suspension was diluted to create a 2 mL working culture of OD_600_ 0.05 in GWM in 15-mL polypropylene culture tubes. Cultures were incubated, with 500 rpm shaking, at 37°C + 5% CO_2_ for 3–5 h prior to antibiotic exposure.

### nuc-aAST time course without enhancing step

Working cultures of *Ng* isolates were prepared as described in “*Ng* culture preparation.” Incubation at 37°C was performed in 100 μL reaction volumes in PCR tube strips on a Bio-Rad C1000 Thermal Cycler. Treated samples consisted of 77.5 μL MHB, 2.5 μL NaHCO_3_ (200 mM), 5 μL DNase I (2 U/μL), 5 μL PEN (20 μg/mL), and 10 μL working *Ng* isolate culture. PEN was replaced with NF-H_2_O in control samples. A 10-μL aliquot of each sample was extracted at 15, 30, 45, 60, 90, and 120 min and diluted 10X in QuickExtract DNA Extraction Solution (Lucigen, Middleton, WI), then heated for 6 min at 65°C followed by 4 min at 98°C on a Bio-Rad C1000 Thermal Cycler. All sample handling following antibiotic exposure was performed using a multichannel pipette; qPCR and calculation of % accessibility were performed as described previously.

### Enhancer use

Working cultures of *Ng* isolates were prepared as described in “*Ng* culture preparation.” Initial exposure was performed by incubating 100 μL control and treated samples at 37°C in PCR tube strips on a Bio-Rad C1000 Thermal Cycler. Treated samples consisted of 75 μL MHB, 5 μL NaHCO_3_ (100 mM), 5 μL DNase I (2 U/μL), 5 μL PEN or CRO (20 μg/mL), and 10 μL working *Ng* isolate culture. Antibiotics were replaced with NF-H_2_O in control samples. After 15 min of incubation, samples were vortexed and quick-spun, and aliquots of all samples were transferred to the enhancement step as described subsequently. After the enhancement step, 5 or 10 μL of all samples was extracted by diluting 10X in QuickExtract DNA Extraction Solution (Lucigen) and heating for 6 min at 65°C followed by 4 min at 98°C on a Bio-Rad C1000 Thermal Cycler. All sample handling following antibiotic exposure was performed using a multichannel pipette; qPCR and calculation of percentage accessibility was performed as described previously.

Osmotic and autolytic enhancing steps were performed in 100 μL volumes. The osmotic enhancing step consisted of 89.75 μL NF-H_2_O, 4.75 μL DNase I (2 U/μL), 0.5 μL CaCl_2_ (100 mM, 0.2-μM filtered), and 5 μL initial exposure samples. The autolytic enhancing step consisted of 75 μL NF-H_2_O, 4.75 μL NaHCO_3_ (100 mM, 0.2-μM filtered), 10 μL Tris (pH 8.5) (500 mM), 4.75 μL DNase I (2 U/μL), 0.5 μL CaCl_2_ (100 mM), and 5 μL of the sample exposed to antibiotic.

All surfactant-enhancing steps were performed in 50 μL volumes with 25 of the 50 μL consisting of initial exposure samples. In the TNP enhancement step, the remaining 25 μL consisted of 1.25 μL DNase I (2 U/μL), 1.25 μL NaHCO_3_ (100 mM), 20 μL MHB, and 2.5 μL TNP (100 mM). In the CHAPS enhancement step, the remaining 25 μL consisted of 1.25 μL DNase I (2 U/μL), 1.25 μL NaHCO_3_ (100 mM), 20 μL MHB, and 2.5 μL CHAPS (200 mM). In the SDS and BAC enhancement steps, the remaining 25 μL consisted of 1.25 μL DNase I (2 U/μL), 1.25 μL NaHCO_3_ (100 mM), 17.5 μL MHB, and either 5 μL SDS (1% w/v) or BAC (1% w/v) respectively.

### nuc-aAST validation

Working cultures were prepared and exposed to antibiotics, and enhancing steps were performed as described for the CHAPS enhancement step in the “enhancer selection” section. Extraction was performed as described previously. Treated samples in the initial exposure step had a final concentration of 1.0 μg/mL PEN, CFM, or CRO. Samples were excluded if the percentage lysis (Eq 2) due to CHAPS was >75%. If the percentage lysis was negative, the value was set to zero before averaging. Three to 13 biological replicates were performed for each isolate-antibiotic combination. Biological replicates included separate antibiotic exposure, control exposure, and no-enhancer controls.

### Preparation of clinical sample suspensions

After initial urine collection by a patient, AHF research staff pipetted an 8–14-mL aliquot into a 15-mL conical tube. For each clinical sample, handling began within 30 min of the sample donation. A 1-mL aliquot of the clinical urine was centrifuged in a 2-mL screw-cap microcentrifuge tube (VWR) for 5 min at 1,000*g* (Eppendorf 5418). The supernatant was then immediately removed and the pellets resuspended in GWM to generate a working suspension. Next, a 320-μL aliquot of the working suspension was added to a mixture of 40 μL 10X DNase I Reaction Buffer (NEB), 20 μL, DNase I (2,000 U/mL) (NEB), and 20 μL Saponin (20% w/v; Cas#8047-15-2, TCI). The suspension was then vortexed, spun in a benchtop microcentrifuge at 2,000*g* for 2–3 sec (Labnet Spectrafuge Mini Microcentrifuge), and placed on a heat block (GeneMate Mini Dry Bath) at 37°C for 15 min. Next, the suspension was vortexed and centrifuged for 5 min at 1,000*g*. The supernatant was removed and the pellet resuspended in a mixture of equal volume and concentration of GWM, DNase I Reaction Buffer, and DNase I, as described previoiusly.

### Clinical sample nuc-aAST

Suspensions of clinical urine samples were prepared as described previously. All suspensions were generated, and the antibiotic-exposure step was initiated within 90 min of sample donation. The initial antibiotic exposure was performed by incubating 50 μL control and treated samples at 37°C in PCR tube strips on a Bio-Rad C1000 Thermal Cycler. Treated samples consisted of 48.8 μL of suspension and 1.25-μL aliquot of PEN or CRO (40 μg/mL). Antibiotics were replaced with NF-H_2_O in control samples. After 30 min of incubation, samples were vortexed and quickly spun on a benchtop microcentrifuge (Labnet) at 2,000*g* for 2–3 sec, and 2.7 μL TNP (100 mM) was added to each sample for the enhancement step. Samples were then immediately vortexed, spun on a benchtop microcentrifuge (Labnet) at 2,000*g* for 2–3 sec, and incubated at 37°C for 3–5 min. After the enhancement step, 20 μL from each sample was extracted by diluting 5X in QuickExtract DNA Extraction Solution (Lucigen) and heating for 6 min at 65°C followed by 4 min at 98°C on a Bio-Rad C1000 Thermal Cycler. All sample handling following antibiotic exposure was performed using a multichannel pipette; qPCR and calculation of percentage accessibility were performed as previously described, with the modification that the qPCR mix included 2 μL of template per 10 μL PCR reaction instead of 1 μL template per 10 μL reaction.

When processing the NA measurements, sample-inclusion criteria were as follows: samples must have had a 16S DNA concentration in the no- antibiotic control tube of less than a Cq of 29 (which translates to approximately 200 copies of 16S DNA/μL DNA extraction or 20 copies/μL in the PCR or LAMP reaction). ASTs from clinical samples with a negative percentage accessibility calculated to be less than −30% accessibility, or those with only one usable replicate out of three, were excluded from analysis.

### MIC testing and creation of clinical isolates

While each of the clinical samples was being run with the nuc-aAST protocol, a 5–10-mL aliquot of the same clinical urine sample was packaged and transported on ice from AHF (Los Angeles, CA) to the laboratory at Caltech (Pasadena, CA). At the Caltech lab, a 50-μL aliquot was plated onto *Neisseria*-selective media (Modified Thayer Martin II [MTMII] Agar; Fisher Scientific) and incubated for 24–72 h at 37°C and 5% CO_2_. Four individual colonies were subcultured onto a fresh MTMII agar plate and incubated for 8–48 h at 37°C and 5% CO_2_. The agar plates were parafilm-sealed, packaged, and shipped overnight via FedEx at ambient temperature for isolation and gold-standard (agar-dilution) MIC testing at the Neisseria Reference Laboratory in Seattle, WA. Agar-dilution MIC testing was performed as previously described.

### Clinical sample nuc-aAST repeated with clinical isolates

After we prepared the clinical isolate and ran the gold-standard agar-dilution MIC test, the isolate was shipped back from the Neisseria Reference Laboratory (Seattle, WA) to the Caltech laboratory (Pasadena, CA). The isolate was then grown according to the methods for “*Ng* culture preparation” previously described. The experimental steps for “Clinical sample preparation” and “Clinical sample nuc-aAST” were repeated using the cell suspension of the isolate instead of the urine sample. The data from these clinical isolates were then compared to the results of the nuc-aAST performed directly on the original clinical urine samples.

### Timed sum of steps

Working cultures of *Ng* isolates used in Fig 4 were prepared as described in “*Ng* culture preparation,” and 1.5 mL of the cultures were pelleted at 2,500*g* for 2.5 min and resuspended in 150 μL normal human urine (Lee Biosciences) prewarmed to 37°C. Initial exposure was performed by incubating 100 μL control and treated samples at 37°C in PCR tube strips on a Bio-Rad C1000 Thermal Cycler. Treated samples consisted of 65 μL MHB, 5 μL NaHCO_3_ (100 mM), 5 μL DNase I (2 U/μL), 5 μL PEN (20 μg/mL), and 20 μL *Ng* isolate suspension in urine. NF-H_2_O was used in place of PEN in control samples. A CHAPS enhancing step was performed as described previously. After the enhancement step, a 20-μL aliquot from each sample was extracted by diluting 5X in QuickExtract DNA Extraction Solution (Lucigen) and heated for 1 min at 65°C followed by 1 min at 98°C on a Bio-Rad C1000 Thermal Cycler. All sample handling following antibiotic exposure was performed using a multichannel pipette. Amplification was then performed using qPCR, ddPCR, or dLAMP. Extractions were diluted 2.5X in NF-H_2_O before use in dLAMP.

### Osmolarity measurements

Osmolarity measurements were performed on a Model 3320 Osmometer (Advanced Instruments, Norwood, MA). The instrument was calibrated with reference standards (Advanced Instruments) prior to experiments. Samples identical to the antibiotic-exposure condition (i.e., media, nuclease, etc.) and samples identical to the osmotic enhancing condition were prepared and measured. The volume that would normally comprise *Ng* culture was replaced with media.

### Statistical analysis

*p*-Values for Fig 2 were calculated using GraphPad Prism 8.0 software from an unpaired, two-tailed *t* test comparing the averages of the three replicates of each susceptible sample to each resistant sample. A significance value of 0.02 was used for statistical significance. ROC plots used for setting susceptibility thresholds in Fig 4 were created using GraphPad Prism 8.0 software. Sensitivity was defined as the proportion of gold-standard susceptible samples correctly identified as susceptible by the nuc-aAST. Specificity was defined as the proportion of gold-standard resistant samples correctly identified as resistant by the nuc-aAST. Statistical analyses for Fig 6 (dLAMP measurements) were performed as published previously [51,106]. As in our previous publication [51], the control and treated concentrations are compared as a ratio for statistical analysis.

$$Concentration\;Ratio=\frac{\lambda_{control}}{\lambda_{treated}}$$ (7)

This concentration ratio is transformed into a percentage change for visualization purposes, but the ratio is assessed for statistical significance. Poisson statistics were used to calculate the confidence interval of the NA concentration for each measurement. The error in the concentration ratio, a term used in the calculation of percentage accessibility, is calculated with standard-error propagation methods:

$$\sigma_{ratio}=\sqrt{{\left(\frac{\sigma_{\lambda_{2}}}{\lambda_{1}}\right)}^{2}+{\left(\frac{\lambda_{2}∙\sigma_{\lambda_{1}}}{\lambda_{1}^{2}}\right)}^{2}}$$ (8)

A one-tailed Z-test, assuming a normal distribution, is used to calculate *p*-values for digital NA concentrations. A threshold value for significance is set as a ratio of 1.22, corresponding to a percentage accessibility of 18%.

$$Z=\frac{ln(l_{control})-ln(1.22\;l_{treated})}{\sqrt{{\sigma^{2}{in}_{(\lambda_{control})+}\sigma }^{2}{in}_{\left(\lambda_{treated}\right)}}}$$ (9)

A significance value of 0.05 was used for statistical significance. The *p*-values to determine significance in dLAMP experiments were computed using Microsoft Excel’s standard normal cumulative distribution function and Z-value.

## Supporting information

S1 Fig DNase is properly inactivated by extraction and heat treatment steps used in this work (see Methods for details). After extraction/inactivation, *Ng* DNA was spiked into the extractions containing the inactivated DNase I and incubated at 37°C. The *Ng* DNA was not degraded, confirming the inactivation of the DNase I enzyme. The concentration of DNase I, the composition of the incubations, and the extraction conditions were all performed under the same conditions as the ASTs. Error bars are 98% confidence intervals for three PCR replicates [55]. Data are in S8 Table. AST, antibiotic susceptibility test; *Ng*, *N*. *gonorrhoeae*.
(TIF)

S2 Fig DNase I remains active in clinical urine samples. (a) The qPCR results for the lambda DNA spike-in for three different urine samples. (b) Percentage of DNA digested calculated from qPCR results with the same equations used previously (see Methods) to calculate percentage lysis. Data are in S9 Table. qPCR, quantitative PCR.
(TIF)

S3 Fig The nuc-aAST using clinical isolates separated by MIC. (a-c) Results of nuc-aAST after 15 min of exposure to (a) PEN, (b) CFM, or (c) CRO. (d-e) nuc-aAST results after exposure to (d) CFM or (e) CRO for 30 min. Each point represents the average for a single isolate run in (at least) biological triplicate for that condition; error bars represent the standard deviation from the biological replicates. All PCR assays were performed in technical triplicate. The dashed line represents the susceptibility threshold, which was set at 26.5% accessibility for 15-min exposures and 46% for 30-min exposures. Data are also plotted in Fig 4. (Data plotted here are in Table S4; experimental data from individual replicates are in S11 Table and S12 Table; MICs are in S1 Table). AST, antibiotic susceptibility test; CFM, cefixime; CRO, ceftriaxone; MIC, minimum inhibitory concentration; nuc-aAST, nuclease-accessibility AST; PEN, penicillin.
(TIF)

S4 Fig ROC curves. ROC curves were generated from the data shown in Fig 4, S4 Table. The FPR is shown on the x-axis and the TPR is shown on the y-axis. The AUC is shown for each plot. **(a-c)** Results of nuc-aAST after 15 min of exposure to **(a)** PEN, **(b)** CFM, or **(c)** CRO. **(d-e)** The nuc-aAST results after exposure to (d) CFM or (e) CRO for 30 min. AUC, area under the curve; CFM, cefixime; CRO, ceftriaxone; FPR, false positive rate; nuc-aAST, nuclease-accessibility antimicrobial susceptibility testing; PEN, penicillin; ROC, receiver operating characteristic; TPR, true positive rate.
(TIF)

S1 Table *N. gonorrhoeae* isolates used in this study. Isolates were obtained from the UW NRL, the CDC AR Isolate Bank, and isolates prepared by the authors from clinical urine samples obtained from consented patients at AHF under IRB #18–0865. The gold-standard MICs for the CDC AR Isolate Bank are from the literature and all other MICs were calculated as described in the Methods. Antibiotics included PEN, CRO, and CFM. AHF, AIDS Healthcare Foundation; AR, Antibiotic Resistance; CDC, Centers for Disease Control; CFM, cefixime; CRO, ceftriaxone; MIC, minimum inhibitory concentration; PEN, penicillin; UW NRL, University of Washington Neisseria Reference Laboratory.
(XLSX)

S2 Table Percentage lysis of *N*. *gonorrhoeae* cells after 5-min exposure to an enhancer. Cqs are measured from qPCR as reported in main methods and the mean of the three PCR triplicates is reported in the table. Eqs 3 and 4 are used to calculate the percentage lysis and the error in that calculation based on error propagation from the standard deviation of qPCR triplicates. Data are plotted in Fig 3A–3F; negative percentages were set to 0 for visualization, as described in Methods. Enhancers included CHAPS at a final concentration of 10 mM; nonionic surfactant TNP at a final concentration of 5 mM and an HLB of 13.1; cationic surfactant BAC at a final concentration of 0.1%; anionic surfactant SDS at a final concentration of 0.01%; Tris buffer (pH 8.5); nuclease-free water. BAC, benzalkonium chloride; CHAPS, zwitterionic surfactant 3-[(3-cholamidopropyl)dimethylammonio]-1-propanesulfonate; HLB, hydrophile-lipophile balance; qPCR, quantitative PCR; SDS, sodium dodecyl sulfate; TNP, TERGITOL NP.
(XLSX)

S3 Table The percentage of *N. gonorrhoeae* DNA accessible after a 15-min ABX exposure (1 μg/mL) followed by a 5-min exposure to an enhancer. The PCR Cq is measured by qPCR (see Methods) and the mean of the PCR triplicates is reported. Eqs 1 and 2 are used to calculate the percentage accessibility, and the error in that calculation is based on the error propagation of the standard deviation of qPCR triplicates. “Treated” indicates the isolate was exposed to an ABX; “control” indicates no ABX exposure. Data are plotted in Fig 3G–3X., negative percentages were set to 0 for visualization, as described in Methods. ABXs included PEN, CRO, and CFM. Isolate categories included S and R. ABX, antibiotic; CFM, cefixime; Cq, quantitation cycle; CRO, ceftriaxone; qPCR, quantitative PCR; R, resistant to ABX; S, susceptible to ABX.
(XLSX)

S4 Table The percentage of *N. gonorrhoeae* DNA accessible after a 15- or 30-min exposure to an ABX and 5-min enhancement with CHAPS. The mean percentage of accessible DNA is calculated from at least three biological replicates of that nuc-aAST condition in clinical isolates (details of individual replicates are shown in S11 Table and S12 Table). Additionally, we report the SEM and SD of the biological replicate nuc-aASTs. Each nuc-aAST used 1 μg/mL of ABXs followed by 5 min of CHAPS as an accessibility “enhancer.” Data are plotted in Fig 4, negative percentage accessibilities were set to 0 for visualization, as described in Methods. ABXs included PEN, CRO, and CFM. Isolates included S and R. ABX, antibiotic; CFM, cefixime; CHAPS, 3-[(3-Cholamidopropyl)dimethylammonio]-1-propanesulfonate; CRO, ceftriaxone; nuc-aAST, nuclease-accessibility antimicrobial susceptibility testing; PEN, penicillin; R, resistant to ABX; S, susceptible to ABX; SD, standard deviation; SEM, standard error of the mean.
(XLSX)

S5 Table The results of nuc-aAST with a dLAMP readout using contrived samples with *Ng* isolates and clinical urine samples positive for *Ng*. Contrived samples are nuc-aASTs performed with clinical Ng isolates spiked into healthy urine; assay conditions are 15-min exposures to 1 μg/mL ABX followed by 5-min exposure to the enhancer CHAPS. One of the three technical replicates from each of the six clinical urine sample nuc-aASTs (Fig 6D and S6 Table) was also run in dLAMP (Fig 6E and S5 Table). Assay conditions were fresh, clinical urine samples, run with 30-min exposures to 1 μg/mL ABX followed by 3–5-min exposure to the enhancer TNP. The concentration of the *Ng* 16S DNA is reported in copies/μL, and *p*-values are calculated as described in the “Statistical analysis” section of the Methods for dLAMP experiments. (Negative percentages were set to 0 for visualization, as described in Methods. In Fig 6E, ABX-susceptible and ABX-intermediate samples are plotted together under the category NR). ABXs included PEN, CRO, and CFM. ABX, antibiotic; CFM, cefixime; CHAPS, 3-[(3-Cholamidopropyl)dimethylammonio]-1-propanesulfonate; CRO, ceftriaxone; dLAMP, digital loop-mediated isothermal amplification; *Ng*, *N*. *gonorrhoeae*; nuc-aAST, nuclease-accessibility antimicrobial susceptibility testing; NR, not resistant; PEN, penicillin; TNP, TERGITOL NP.
(XLSX)

S6 Table nuc-aAST data for clinical urine samples and isolates from the samples. All nuc-aASTs were run with a 30-min ABX exposure and 3–5 min of TNP enhancement step. The internal isolate number and clinical sample name corresponds to the MIC values in S1 Table. The sample number corresponds to the clinical sample number reported in Fig 5D. The sample type refers to if the nuc-aAST results come from an assay run directly from the clinical urine sample or on the isolate prepared from that urine sample. Technical replicates are parallel nuc-aASTs run for each condition. The percentage accessibility is calculated from the qPCR measurements, and the error is propagated according to Eqs 2 and 3, as described for previous calculations. ABXs included PEN and CRO. ABX, antibiotic; CRO, ceftriaxone; MIC, minimum inhibitory concentration; nuc-aAST, nuclease-accessibility antimicrobial susceptibility testing; qPCR, quantitative PCR; PEN, penicillin; TNP, TERGITOL NP.
(XLSX)

S7 Table nuc-aAST summary of samples and isolates from the samples. The data are summarized with the mean percentage accessibility calculated from the technical replicate nuc-aASTs (mean percentages accessibility are shown in Fig 5D). Additionally, the SEM and SD are reported from the technical replicate nuc-aASTs. The technical replicates in this table are only the replicates that met our criteria for inclusion as described in the Methods. ABXs included PEN and CRO. ABX, antibiotic; CRO, ceftriaxone; nuc-aAST, nuclease-accessibility antimicrobial susceptibility testing; PEN, penicillin; SD, standard deviation; SEM, standard error of the mean.
(XLSX)

S8 Table DNase I inactivation data. Data are plotted in S1 Fig. Mean Cq is computed from qPCR triplicates. Error bars are 98% confidence intervals for three PCR replicates [55]. Cq, quantitation cycle; qPCR, quantitative PCR.
(XLSX)

S9 Table DNA digestion of the lambda spike-in. Mean Cq is calculated from PCR triplicates and error is calculated (as described in main Methods) for PCR triplicates. Percentage DNA digestion is calculated for each clinical sample from the samples with and without DNase I. The error in the PCR measurements is propagated for calculating the error bar in the percentage digestion of the spike-in DNA. Data are shown in S2 Fig. Cq, quantitation cycle.
(XLSX)

S10 Table Percentage accessibility of DNA over time without an enhancer. Data are plotted in Fig 2. ABX, antibiotic; R, resistant to ABX; S, susceptible to ABX.
(XLSX)

S11 Table Each biological replicate nuc-aAST of Ng DNA accessible after a 15-min exposure to an ABX and 5-min enhancement with CHAPS. Replicate numbers refer to which biological replicate experiment the data are from. The PCR Cq is measured by qPCR (see Methods) and the mean of the PCR triplicates is reported. Eq 1 is used to calculate the percentage accessibility, and Eq 3 is used to calculate the percentage lysis. “Treated” indicates the isolate was exposed to an ABX; “control” indicates no ABX exposure. Data are used to calculate mean percentage accessibility for each isolate, which are plotted in Fig 4A–4C and S3 Fig and shown in S4 Table. ABXs included PEN, CRO, and CFM. Susceptibility was indicated by S, R, or I. ABX, antibiotic; CFM, cefixime; CHAPS, 3-[(3-Cholamidopropyl)dimethylammonio]-1-propanesulfonate; Cq, quantitation cycle; CRO, ceftriaxone; I, intermediate MIC; MIC, minimum inhibitory concentration; Ng, N. gonorrhoeae; nuc-aAST, nuclease-accessibility antimicrobial susceptibility testing; PEN, penicillin; qPCR, quantitative PCR; R, resistant to ABXs; S, susceptible to ABXs.
(XLSX)

S12 Table Each biological replicate nuc-aAST of *Ng* DNA accessible after a 30-min exposure to an ABX and 5-min enhancement with CHAPS. Replicate numbers refer to which biological replicate experiment the data are from. The PCR Cq is measured by qPCR (see Methods), and the mean of the PCR triplicates is reported. Eq 1 is used to calculate the percentage accessibility, and Eq 3 is used to calculate the percentage lysis. “Treated” indicates the isolate was exposed to an ABX; “control” indicates no ABX exposure. Data are used to calculate mean percentage accessibility for each isolate, which are plotted in Fig 4D and 4E and S3 Fig and shown in S4 Table. ABXs included PEN, CRO, and CFM. Susceptibility was indicated by S or R. ABX, antibiotic; CFM, cefixime; CHAPS, 3-[(3-Cholamidopropyl)dimethylammonio]-1-propanesulfonate; Cq, quantitation cycle; CRO, ceftriaxone; *Ng*, *N*. *gonorrhoeae*; nuc-aAST, nuclease-accessibility antimicrobial susceptibility testing; PEN, penicillin; qPCR, quantitative PCR; R, resistant to ABX; S, susceptible to ABX.
(XLSX)

S1 Text Supplementary materials and methods and a detailed statement of author contributions. (PDF)

S1 Data A representative subset of fluorescence values from a digital LAMP AST run on a susceptible *N*. *gonorrhoeae* isolate in the control (no antibiotic) treatment. A representative AST from one of the contrived samples using isolates was selected and the LAMP amplification curves from the first 100 positive wells are shown. These curves are plotted in Fig 6B. See also S2–S4 Data for the complete dataset used in Fig 6B. AST, antibiotic susceptibility test; LAMP, loop-mediated isothermal amplification.
(XLSX)

S2 Data A representative subset of fluorescence values from a digital LAMP AST run on a susceptible *N*. *gonorrhoeae* isolate in the treated (penicillin) treatment. These are representative wells to show LAMP amplification curves. These curves are plotted in Fig 6B. A representative AST from one of the contrived samples using isolates was selected. See also S1, S3 and S4 Data for the complete dataset used in Fig 6B. AST, antibiotic susceptibility test; LAMP, loop-mediated isothermal amplification.
(XLSX)

S3 Data A representative subset of fluorescence values from a digital LAMP AST run on a resistant *N*. *gonorrhoeae* isolate in the control (no antibiotic) treatment. These are representative wells to show LAMP amplification curves. These curves are plotted in Fig 6B. A representative AST from one of the contrived samples using isolates was selected. See also S1, S2 and S4 Data for the complete dataset used in Fig 6B. AST, antibiotic susceptibility test; LAMP, loop-mediated isothermal amplification.
(XLSX)

S4 Data A representative subset of fluorescence values from a digital LAMP AST run on a resistant *N*. *gonorrhoeae* isolate in the treated (penicillin) treatment. These are representative wells to show LAMP amplification curves. These curves are plotted in Fig 6B. A representative AST from one of the contrived samples using isolates was selected. See also S1–S3 Data for the complete dataset used in Fig 6B. AST, antibiotic susceptibility test; LAMP, loop-mediated isothermal amplification.
(XLSX)

## Abbreviations

AHF: AIDS Healthcare Foundation
AST: antibiotic/antimicrobial susceptibility test/testing
AUC: area under the curve
AZM: azithromycin
BAC: benzalkonium chloride
CDC: Centers for Disease Control and Prevention
CFM: cefixime
CHAPS: 3-[(3-Cholamidopropyl)dimethylammonio]-1-propanesulfonate
CIP: ciprofloxacin
CRO: ceftriaxone
dLAMP: digital loop-mediated isothermal amplification
GWM: Graver-Wade medium
ID: identification
MIC: minimum inhibitory concentration
NA: nucleic acid
NF-H_2_O: nuclease-free water
*Ng*: *Neisseria gonorrhoeae*
NR: not resistant
nuc-aAST: nuclease-accessibility AST
OM: outer membrane
PEN: penicillin
PEN-I: penicillin-intermediate
POC: point of care
qPCR: quantitative PCR
RFU: relative fluorescence units
ROC: receiver operating characteristic
SDS: sodium dodecyl sulfate
STI: sexually transmitted infection
TET: tetracycline
TNP: TERGITOL NP
UTI: urinary tract infection
WHO: World Health Organization

## References

[1] CDC. Sexually Transmitted Disease Surveillance 2017. Available from: https://www.cdc.gov/std/stats17/default.htm. [cited 2020 Mar 3].

[2] Rowley J, Vander Hoorn S, Korenromp E, Low N, Unemo M, Abu-Raddad L, et al Chlamydia, gonorrhoea, trichomoniasis and syphilis: global prevalence and incidence estimates, 2016. Bull WHO. 2019;BLT.18.228486 10.2471/BLT.18.228486 PMC6653813 31384073

[3] Quillin SJ, Seifert HS. Neisseria gonorrhoeae host adaptation and pathogenesis. Nat Rev Microbiol. 2018;16(4):226–40. 10.1038/nrmicro.2017.169 29430011 PMC6329377

[4] Chesson HW, Kirkcaldy RD, Gift TL, Owusu-Edusei K Jr., Weinstock HS. An Illustration of the Potential Health and Economic Benefits of Combating Antibiotic-Resistant Gonorrhea. Sex Transm Dis. 2018;45(4):250–3. 10.1097/OLQ.0000000000000725 29465709 PMC6724164

[5] CDC. Antibiotic resistance threats in the United States, 2019. Atlanta: US Department of Health and Human Services, CDC; 2019. doi: CS239559-B

[6] Workowski KA, Bolan GA. Sexually transmitted diseases treatment guidelines, 2015. MMWR Recomm Rep. 2015;64(RR-03):1. Available from: https://www.cdc.gov/std/tg2015/tg-2015-print.pdf. [cited 2020 Mar 3]. 26042815 PMC5885289

[7] WHO. WHO Guidelines for the treatment of Neisseria gonorrhoeae. 2016. 27512795

[8] Newman LM, Moran JS, Workowski KA. Update on the management of gonorrhea in adults in the United States. Clin Infect Dis. 2007;44 Suppl 3:S84–101. 10.1086/511422 17342672

[9] Tapsall J; WHO. Antimicrobrial Resistance in Neisseria gonorrhoeae. World Health Organization, Department of Communicable Disease Surveillance and Response; 2001.

[10] Fifer H, Natarajan U, Jones L, Alexander S, Hughes G, Golparian D, et al Failure of Dual Antimicrobial Therapy in Treatment of Gonorrhea. N Engl J Med. 2016;374(25):2504–6. 10.1056/NEJMc1512757 27332921

[11] Whiley DM, Jennison A, Pearson J, Lahra MM. Genetic characterisation of Neisseria gonorrhoeae resistant to both ceftriaxone and azithromycin. Lancet Infect Dis. 2018;18(7):717–8. 10.1016/S1473-3099(18)30340-2 29976521

[12] Eyre DW, Sanderson ND, Lord E, Regisford-Reimmer N, Chau K, Barker L, et al Gonorrhoea treatment failure caused by a Neisseria gonorrhoeae strain with combined ceftriaxone and high-level azithromycin resistance, England, February 2018. Euro Surveill. 2018;23(27). 10.2807/1560-7917.ES.2018.23.27.1800323 29991383 PMC6152157

[13] CDC. Antibiotic Resistance Threats in the United States, 2013. 2013 [cited 2018 Dec 3]. Available from: https://www.cdc.gov/drugresistance/pdf/ar-threats-2013-508.pdf.

[14] WHO. Global Priority List of Antibiotic-resistant Bacteria to Guide Research, Discovery, and Development of New Antibiotics. 2017 [cited 2018 Dec 3]. Available from: https://www.who.int/medicines/publications/global-priority-list-antibiotic-resistant-bacteria/en/.

[15] Bolan GA, Sparling PF, Wasserheit JN. The emerging threat of untreatable gonococcal infection. N Engl J Med. 2012;366(6):485–7. 10.1056/NEJMp1112456 22316442

[16] Wi T, Lahra MM, Ndowa F, Bala M, Dillon J-AR, Ramon-Pardo P, et al Antimicrobial resistance in Neisseria gonorrhoeae: Global surveillance and a call for international collaborative action. PLoS Med. 2017;14(7):e1002344 10.1371/journal.pmed.1002344 28686231 PMC5501266

[17] WHO. Global Action Plan to Control the Spread of and Impact of Antimicrobial Resistance in Neisseria gonorrhoeae. 2012.

[18] Weston EJ, Wi T, Papp J. Strengthening Global Surveillance for Antimicrobial Drug-Resistant Neisseria gonorrhoeae through the Enhanced Gonococcal Antimicrobial Surveillance Program. Emerg Infect Dis. 2017;23(13). 10.3201/eid2313.170443 29155673 PMC5711314

[19] WHO. No time to wait: Securing the future from drug-resistant infections. Report to the Secretary-General of the United Nations. 2019.

[20] Rahimi F, Goire N, Guy R, Kaldor JM, Ward J, Nissen MD, et al Direct urine polymerase chain reaction for chlamydia and gonorrhoea: a simple means of bringing high-throughput rapid testing to remote settings? Sex Health. 2013;10(4):299–304. 10.1071/SH12108 23639791

[21] Cho S, Park TS, Nahapetian TG, Yoon JY. Smartphone-based, sensitive microPAD detection of urinary tract infection and gonorrhea. Biosens Bioelectron. 2015;74:601–11. 10.1016/j.bios.2015.07.014 26190472

[22] Gootenberg JS, Abudayyeh OO, Kellner MJ, Joung J, Collins JJ, Zhang F. Multiplexed and portable nucleic acid detection platform with Cas13, Cas12a, and Csm6. Science. 2018;360(6387):439–44. 10.1126/science.aaq0179 29449508 PMC5961727

[23] binx. io system for clinical point of care. 2018 [cited 2020 Mar 3]. Available from: https://mybinxhealth.com/point-of-care/.

[24] Gaydos CA, Van Der Pol B, Jett-Goheen M, Barnes M, Quinn N, Clark C, et al Performance of the Cepheid CT/NG Xpert Rapid PCR Test for Detection of Chlamydia trachomatis and Neisseria gonorrhoeae. J Clin Microbiol. 2013;51(6):1666–72. 10.1128/JCM.03461-12 23467600 PMC3716060

[25] Allan-Blitz LT, Wang X, Klausner JD. Wild-Type Gyrase A Genotype of Neisseria gonorrhoeae Predicts In Vitro Susceptibility to Ciprofloxacin: A Systematic Review of the Literature and Meta-Analysis. Sex Transm Dis. 2017;44(5):261–5. 10.1097/OLQ.0000000000000591 28407640 PMC5407314

[26] Li Z, Yokoi S, Kawamura Y, Maeda S, Ezaki T, Deguchi T. Rapid detection of quinolone resistance-associated gyrA mutations in Neisseria gonorrhoeae with a LightCycler. J Infect Chemother. 2002;8(2):145–50. 10.1007/s101560200025 12111567

[27] Balashov S, Mordechai E, Adelson ME, Gygax SE. Multiplex bead suspension array for screening Neisseria gonorrhoeae antibiotic resistance genetic determinants in noncultured clinical samples. J Mol Diagn. 2013;15(1):116–29. 10.1016/j.jmoldx.2012.08.005 23159594

[28] Dona V, Smid JH, Kasraian S, Egli-Gany D, Dost F, Imeri F, et al Mismatch Amplification Mutation Assay-Based Real-Time PCR for Rapid Detection of Neisseria gonorrhoeae and Antimicrobial Resistance Determinants in Clinical Specimens. J Clin Microbiol. 2018;56(9). 10.1128/JCM.00365-18 29950339 PMC6113480

[29] Buckley C, Trembizki E, Donovan B, Chen M, Freeman K, Guy R, et al Real-time PCR detection of Neisseria gonorrhoeae susceptibility to penicillin. J Antimicrob Chemother. 2016;71(11):3090–5. 10.1093/jac/dkw291 27494921

[30] Wong LK, Hemarajata P, Soge OO, Humphries RM, Klausner JD. Real-Time PCR Targeting the penA Mosaic XXXIV Type for Prediction of Extended-Spectrum-Cephalosporin Susceptibility in Clinical Neisseria gonorrhoeae Isolates. Antimicrob Agents Chemother. 2017;61(11). 10.1128/AAC.01339-17 28848021 PMC5655115

[31] Davies J, Davies D. Origins and evolution of antibiotic resistance. Microbiol Mol Biol Rev. 2010;74(3):417–33. 10.1128/MMBR.00016-10 20805405 PMC2937522

[32] CDC. Agar Dilution Antimicrobial Susceptibility Testing. 2013 [cited 2018 Oct 31]. Available from: https://www.cdc.gov/std/gonorrhea/lab/agar.htm.

[33] Foerster S, Desilvestro V, Hathaway LJ, Althaus CL, Unemo M. A new rapid resazurin-based microdilution assay for antimicrobial susceptibility testing of Neisseria gonorrhoeae. J Antimicrob Chemother. 2017;72(7):1961–8. 10.1093/jac/dkx113 28431096 PMC5890744

[34] Singh V, Bala M, Kakran M, Ramesh V. Comparative assessment of CDS, CLSI disc diffusion and Etest techniques for antimicrobial susceptibility testing of Neisseria gonorrhoeae: a 6-year study. BMJ Open. 2012;2(4):e000969 10.1136/bmjopen-2012-000969 22761285 PMC3391364

[35] Siedner MJ, Pandori M, Castro L, Barry P, Whittington WL, Liska S, et al Real-time PCR assay for detection of quinolone-resistant Neisseria gonorrhoeae in urine samples. J Clin Microbiol. 2007;45(4):1250–4. 10.1128/JCM.01909-06 17267635 PMC1865802

[36] Morse SA, Hebeler BH. Effect of pH on the growth and glucose metabolism of Neisseria gonorrhoeae. Infect Immun. 1978;21(1):87–95. 30699 10.1128/iai.21.1.87-95.1978 PMC421961

[37] Tobiason DM, Seifert HS. The obligate human pathogen, Neisseria gonorrhoeae, is polyploid. PLoS Biol. 2006;4(6):1069–78. 10.1371/journal.pbio.0040185 16719561 PMC1470461

[38] Wade JJ, Graver MA. A fully defined, clear and protein-free liquid medium permitting dense growth of Neisseria gonorrhoeae from very low inocula. FEMS Microbiol Lett. 2007;273(1):35–7. 10.1111/j.1574-6968.2007.00776.x 17559396

[39] Sadiq ST, Mazzaferri F, Unemo M. Rapid accurate point-of-care tests combining diagnostics and antimicrobial resistance prediction for Neisseria gonorrhoeae and Mycoplasma genitalium. Sex Transm Infect. 2017;93(S4):S65–S8. 10.1136/sextrans-2016-053072 28684610

[40] Fingerhuth SM, Low N, Bonhoeffer S, Althaus CL. Detection of antibiotic resistance is essential for gonorrhoea point-of-care testing: a mathematical modelling study. BMC Med. 2017;15(1):142 10.1186/s12916-017-0881-x 28747205 PMC5530576

[41] Tuite AR, Gift TL, Chesson HW, Hsu K, Salomon JA, Grad YH. Impact of Rapid Susceptibility Testing and Antibiotic Selection Strategy on the Emergence and Spread of Antibiotic Resistance in Gonorrhea. J Infect Dis. 2017;216(9):1141–9. 10.1093/infdis/jix450 28968710 PMC5853443

[42] Turner KM, Christensen H, Adams EJ, McAdams D, Fifer H, McDonnell A, et al Analysis of the potential for point-of-care test to enable individualised treatment of infections caused by antimicrobial-resistant and susceptible strains of Neisseria gonorrhoeae: a modelling study. BMJ Open. 2017;7(6):e015447 10.1136/bmjopen-2016-015447 28615273 PMC5734280

[43] Kirkcaldy RD, Harvey A, Papp JR, Del Rio C, Soge OO, Holmes KK, et al Neisseria gonorrhoeae Antimicrobial Susceptibility Surveillance—The Gonococcal Isolate Surveillance Project, 27 Sites, United States, 2014. MMWR Surveill Summ. 2016;65(7):1–19. 10.15585/mmwr.ss6507a1 27414503

[44] Kirkcaldy RD, Bartoces MG, Soge OO, Riedel S, Kubin G, Del Rio C, et al Antimicrobial Drug Prescription and Neisseria gonorrhoeae Susceptibility, United States, 2005–2013. Emerg Infect Dis. 2017;23(10):1657–63. 10.3201/eid2310.170488 28930001 PMC5621530

[45] Allen VG, Mitterni L, Seah C, Rebbapragada A, Martin IE, Lee C, et al Neisseria gonorrhoeae treatment failure and susceptibility to cefixime in Toronto, Canada. JAMA. 2013;309(2):163–70. 10.1001/jama.2012.176575 23299608

[46] Katz AR, Komeya AY, Kirkcaldy RD, Whelen AC, Soge OO, Papp JR, et al Cluster of Neisseria gonorrhoeae Isolates With High-level Azithromycin Resistance and Decreased Ceftriaxone Susceptibility, Hawaii, 2016. Clin Infect Dis. 2017;65(6):918–23. 10.1093/cid/cix485 28549097 PMC6748320

[47] Papp JR, Abrams AJ, Nash E, Katz AR, Kirkcaldy RD, O'Connor NP, et al Azithromycin Resistance and Decreased Ceftriaxone Susceptibility in Neisseria gonorrhoeae, Hawaii, USA. Emerg Infect Dis. 2017;23(5):830–2. 10.3201/eid2305.170088 28418303 PMC5403062

[48] Piddock LJ. Assess drug-resistance phenotypes, not just genotypes. Nat Microbiol. 2016;1(8):16120 10.1038/nmicrobiol.2016.120 27573119

[49] Hicks JM, Haeckel R, Price CP, Lewandrowski K, Wu AHB. Recommendations and opinions for the use of point-of-care testing for hospitals and primary care: summary of a 1999 symposium. Clin Chim Acta. 2001;303(1–2):1–17. 10.1016/s0009-8981(00)00400-9 11163017

[50] Marston HD, Dixon DM, Knisely JM, Palmore TN, Fauci AS. Antimicrobial Resistance. JAMA. 2016;316(11):1193–204. 10.1001/jama.2016.11764 27654605

[51] Schoepp NG, Schlappi TS, Curtis MS, Butkovich SS, Miller S, Humphries RM, et al Rapid pathogen-specific phenotypic antibiotic susceptibility testing using digital LAMP quantification in clinical samples. Sci Transl Med. 2017;9(410). 10.1126/scitranslmed.aal3693 28978750 PMC6765391

[52] Mezger A, Gullberg E, Goransson J, Zorzet A, Herthnek D, Tano E, et al A general method for rapid determination of antibiotic susceptibility and species in bacterial infections. J Clin Microbiol. 2015;53(2):425–32. 10.1128/JCM.02434-14 25411178 PMC4298551

[53] Mach KE, Mohan R, Baron EJ, Shih MC, Gau V, Wong PK, et al A biosensor platform for rapid antimicrobial susceptibility testing directly from clinical samples. J Urol. 2011;185(1):148–53. 10.1016/j.juro.2010.09.022 21074208 PMC4051414

[54] Halford C, Gonzalez R, Campuzano S, Hu B, Babbitt JT, Liu J, et al Rapid antimicrobial susceptibility testing by sensitive detection of precursor rRNA using a novel electrochemical biosensing platform. Antimicrob Agents Chemother. 2013;57(2):936–43. 10.1128/AAC.00615-12 23229486 PMC3553690

[55] Schoepp NG, Khorosheva EM, Schlappi TS, Curtis MS, Humphries RM, Hindler JA, et al Digital Quantification of DNA Replication and Chromosome Segregation Enables Determination of Antimicrobial Susceptibility after only 15 Minutes of Antibiotic Exposure. Angew Chem Int Ed Engl. 2016;55(33):9557–61. 10.1002/anie.201602763 27357747 PMC5215780

[56] Khazaei T, Barlow JT, Schoepp NG, Ismagilov RF. RNA markers enable phenotypic test of antibiotic susceptibility in Neisseria gonorrhoeae after 10 minutes of ciprofloxacin exposure. Sci Rep. 2018;8(1):11606 10.1038/s41598-018-29707-w 30072794 PMC6072703

[57] Chen L, Shin DJ, Zheng S, Melendez JH, Gaydos CA, Wang T-H. Direct-qPCR Assay for Coupled Identification and Antimicrobial Susceptibility Testing of Neisseria gonorrhoeae. ACS Infectious Diseases. 2018;4(9):1377–84. 10.1021/acsinfecdis.8b00104 29999304 PMC6138521

[58] Unemo M, Shafer WM. Antimicrobial resistance in Neisseria gonorrhoeae in the 21st century: past, evolution, and future. Clin Microbiol Rev. 2014;27(3):587–613. 10.1128/CMR.00010-14 24982323 PMC4135894

[59] WHO. WHO Guidelines for the treatment of Neisseria gonorrhoeae. 2016 [cited 2020 Mar 3]. Available from: https://www.who.int/reproductivehealth/publications/rtis/gonorrhoea-treatment-guidelines/en/. 27512795

[60] EUCAST. European Committee on Antimicrobial Susceptibility Testing: Breakpoint Tables for Interpretation of MICs and Zone Diameters (ver. 7.1). 2017 [cited 2020 Mar 3]. Available from: http://www.eucast.org/clinical_breakpoints/.

[61] Kohanski MA, Dwyer DJ, Collins JJ. How antibiotics kill bacteria: from targets to networks. Nat Rev Microbiol. 2010;8(6):423–35. 10.1038/nrmicro2333 20440275 PMC2896384

[62] Rojas ER, Billings G, Odermatt PD, Auer GK, Zhu L, Miguel A, et al The outer membrane is an essential load-bearing element in Gram-negative bacteria. Nature. 2018;559(7715):617–21. 10.1038/s41586-018-0344-3 30022160 PMC6089221

[63] Wegener WS, Hebeler BH, Morse SA. Cell envelope of Neisseria gonorrhoeae: penicillin enhancement of peptidoglycan hydrolysis. Infect Immun. 1977;18(3):717–25. 22492 10.1128/iai.18.3.717-725.1977 PMC421294

[64] Dillard JP, Seifert HS. A peptidoglycan hydrolase similar to bacteriophage endolysins acts as an autolysin in Neisseria gonorrhoeae. Mol Microbiol. 1997;25(5):893–901. 10.1111/j.1365-2958.1997.mmi522.x 9364915

[65] Zgurskaya HI, Lopez CA, Gnanakaran S. Permeability Barrier of Gram-Negative Cell Envelopes and Approaches To Bypass It. ACS Infect Dis. 2015;1(11):512–22. 10.1021/acsinfecdis.5b00097 26925460 PMC4764994

[66] Silhavy TJ, Kahne D, Walker S. The bacterial cell envelope. Cold Spring Harb Perspect Biol. 2010;2(5):a000414 10.1101/cshperspect.a000414 20452953 PMC2857177

[67] Demchick P, Koch AL. The permeability of the wall fabric of Escherichia coli and Bacillus subtilis. J Bacteriol. 1996;178(3):768–73. 10.1128/jb.178.3.768-773.1996 8550511 PMC177723

[68] Dijkstra AJ, Keck W. Peptidoglycan as a barrier to transenvelope transport. J Bacteriol. 1996;178(19):5555–62. 10.1128/jb.178.19.5555-5562.1996 8824596 PMC178390

[69] Pink D, Moeller J, Quinn B, Jericho M, Beveridge T. On the architecture of the gram-negative bacterial murein sacculus. J Bacteriol. 2000;182(20):5925–30. 10.1128/jb.182.20.5925-5930.2000 11004199 PMC94722

[70] Harrison ST. Bacterial cell disruption: a key unit operation in the recovery of intracellular products. Biotechnol Adv. 1991;9(2):217–40. 10.1016/0734-9750(91)90005-g 14548738

[71] Vazquez-Laslop N, Lee H, Hu R, Neyfakh AA. Molecular sieve mechanism of selective release of cytoplasmic proteins by osmotically shocked Escherichia coli. J Bacteriol. 2001;183(8):2399–404. 10.1128/JB.183.8.2399-2404.2001 11274096 PMC95153

[72] van den Bogaart G, Hermans N, Krasnikov V, Poolman B. Protein mobility and diffusive barriers in Escherichia coli: consequences of osmotic stress. Mol Microbiol. 2007;64(3):858–71. 10.1111/j.1365-2958.2007.05705.x 17462029

[73] Hebeler BH, Young FE. Autolysis of Neisseria gonorrhoeae. J Bacteriol. 1975;122(2):385–92. 236277 10.1128/jb.122.2.385-392.1975 PMC246068

[74] Wegener WS, Hebeler BH, Morse SA. Cell envelope of Neisseria gonorrhoeae: relationship between autolysis in buffer and the hydrolysis of peptidoglycan. Infect Immun. 1977;18(1):210–9. 20406 10.1128/iai.18.1.210-219.1977 PMC421215

[75] Felix H. Permeabilized cells. Anal Biochem. 1982;120(2):211–34. 10.1016/0003-2697(82)90340-2 6178313

[76] Nikaido H. Molecular basis of bacterial outer membrane permeability revisited. Microbiol Mol Biol Rev. 2003;67(4):593–656. 10.1128/MMBR.67.4.593-656.2003 14665678 PMC309051

[77] Zhao S, Duncan M, Tomberg J, Davies C, Unemo M, Nicholas RA. Genetics of chromosomally mediated intermediate resistance to ceftriaxone and cefixime in Neisseria gonorrhoeae. Antimicrob Agents Chemother. 2009;53(9):3744–51. 10.1128/AAC.00304-09 19528266 PMC2737842

[78] Foerster S, Unemo M, Hathaway LJ, Low N, Althaus CL. Time-kill curve analysis and pharmacodynamic modelling for in vitro evaluation of antimicrobials against Neisseria gonorrhoeae. BMC Microbiol. 2016;16:216 10.1186/s12866-016-0838-9 27639378 PMC5027106

[79] Zou KH, O'Malley AJ, Mauri L. Receiver-operating characteristic analysis for evaluating diagnostic tests and predictive models. Circulation. 2007;115(5):654–7. 10.1161/CIRCULATIONAHA.105.594929 17283280

[80] Grad YH, Harris SR, Kirkcaldy RD, Green AG, Marks DS, Bentley SD, et al Genomic Epidemiology of Gonococcal Resistance to Extended-Spectrum Cephalosporins, Macrolides, and Fluoroquinolones in the United States, 2000–2013. J Infect Dis. 2016;214(10):1579–87. 10.1093/infdis/jiw420 27638945 PMC5091375

[81] Wadsworth CB, Sater MRA, Bhattacharyya RP, Grad YH. Impact of Species Diversity on the Design of RNA-Based Diagnostics for Antibiotic Resistance in Neisseria gonorrhoeae. Antimicrob Agents Chemother. 2019;63(8):e00549–19. 10.1128/AAC.00549-19 31138575 PMC6658776

[82] Gianecini RA, Golparian D, Zittermann S, Litvik A, Gonzalez S, Oviedo C, et al Genome-based epidemiology and antimicrobial resistance determinants of Neisseria gonorrhoeae isolates with decreased susceptibility and resistance to extended-spectrum cephalosporins in Argentina in 2011–16. J Antimicrob Chemother. 2019/3/02 ed2019. 10.1093/jac/dkz054 30820563

[83] Baumann E, Stoya G, Volkner A, Richter W, Lemke C, Linss W. Hemolysis of human erythrocytes with saponin affects the membrane structure. Acta Histochem. 2000;102(1):21–35. 10.1078/0065-1281-00534 10726162

[84] Charalampous T, Kay GL, Richardson H, Aydin A, Baldan R, Jeanes C, et al Nanopore metagenomics enables rapid clinical diagnosis of bacterial lower respiratory infection. Nat Biotechnol. 2019;37(7):783–92. 10.1038/s41587-019-0156-5 31235920

[85] Murata-Kamiya N, Kikuchi K, Hayashi T, Higashi H, Hatakeyama M. Helicobacter pylori exploits host membrane phosphatidylserine for delivery, localization, and pathophysiological action of the CagA oncoprotein. Cell Host Microbe. 2010;7(5):399–411. 10.1016/j.chom.2010.04.005 20478541

[86] Rolando JC, Jue E, Schoepp NG, Ismagilov RF. Real-Time, Digital LAMP with Commercial Microfluidic Chips Reveals the Interplay of Efficiency, Speed, and Background Amplification as a Function of Reaction Temperature and Time. Anal Chem. 2018 10.1021/acs.analchem.8b04324 30565936 PMC6322147

[87] Selck DA, Ismagilov RF. Instrument for Real-Time Digital Nucleic Acid Amplification on Custom Microfluidic Devices. PLoS ONE. 2016;11(10):e0163060 10.1371/journal.pone.0163060 27760148 PMC5070811

[88] Lee SG, Lee H, Jeong SH, Yong D, Chung GT, Lee YS, et al Various penA mutations together with mtrR, porB and ponA mutations in Neisseria gonorrhoeae isolates with reduced susceptibility to cefixime or ceftriaxone. J Antimicrob Chemother. 2010;65(4):669–75. 10.1093/jac/dkp505 20093260 PMC2837549

[89] FDA. Evaluation of Automatic Class III Designation for T2Candida Panel and T2Dx Instrument. 2014.

[90] Barczak AK, Gomez JE, Kaufmann BB, Hinson ER, Cosimi L, Borowsky ML, et al RNA signatures allow rapid identification of pathogens and antibiotic susceptibilities. Proc Natl Acad Sci U S A. 2012;109(16):6217–22. 10.1073/pnas.1119540109 22474362 PMC3341018

[91] Avesar J, Rosenfeld D, Truman-Rosentsvit M, Ben-Arye T, Geffen Y, Bercovici M, et al Rapid phenotypic antimicrobial susceptibility testing using nanoliter arrays. Proceedings of the National Academy of Sciences. 2017;114(29):E5787 10.1073/pnas.1703736114 28652348 PMC5530678

[92] Liu T, Lu Y, Gau V, Liao JC, Wong PK. Rapid antimicrobial susceptibility testing with electrokinetics enhanced biosensors for diagnosis of acute bacterial infections. Ann Biomed Eng. 2014;42(11):2314–21. 10.1007/s10439-014-1040-6 24889716 PMC4206609

[93] Premasiri WR, Chen Y, Williamson PM, Bandarage DC, Pyles C, Ziegler LD. Rapid urinary tract infection diagnostics by surface-enhanced Raman spectroscopy (SERS): identification and antibiotic susceptibilities. Anal Bioanal Chem. 2017;409(11):3043–54. 10.1007/s00216-017-0244-7 28235996

[94] Baltekin O, Boucharin A, Tano E, Andersson DI, Elf J. Antibiotic susceptibility testing in less than 30 min using direct single-cell imaging. Proc Natl Acad Sci U S A. 2017;114(34):9170–5. 10.1073/pnas.1708558114 28790187 PMC5576829

[95] Bhattacharyya RP, Bandyopadhyay N, Ma P, Son SS, Liu J, He LL, et al Simultaneous detection of genotype and phenotype enables rapid and accurate antibiotic susceptibility determination. Nat Med. 2019;25(12):1858–64. 10.1038/s41591-019-0650-9 31768064 PMC6930013

[96] Li L, Du W, Ismagilov R. User-loaded SlipChip for equipment-free multiplexed nanoliter-scale experiments. J Am Chem Soc. 2010;132(1):106–11. 10.1021/ja908555n 20000708 PMC2802657

[97] Shen F, Du W, Davydova EK, Karymov MA, Pandey J, Ismagilov RF. Nanoliter multiplex PCR arrays on a SlipChip. Anal Chem. 2010;82(11):4606–12. 10.1021/ac1007249 20446698 PMC2916686

[98] Shen F, Sun B, Kreutz JE, Davydova EK, Du W, Reddy PL, et al Multiplexed quantification of nucleic acids with large dynamic range using multivolume digital RT-PCR on a rotational SlipChip tested with HIV and hepatitis C viral load. J Am Chem Soc. 2011;133(44):17705–12. 10.1021/ja2060116 21995644 PMC3216675

[99] Newman L, Rowley J, Vander Hoorn S, Wijesooriya NS, Unemo M, Low N, et al Global Estimates of the Prevalence and Incidence of Four Curable Sexually Transmitted Infections in 2012 Based on Systematic Review and Global Reporting. PLoS ONE. 2015;10(12):e0143304 10.1371/journal.pone.0143304 26646541 PMC4672879

[100] Schoepp NG, Liaw EJ, Winnett A, Savela ES, Garner OB, Ismagilov RF. Differential Differential DNA accessibility to polymerase enables 30-minute phenotypic β-lactam antibiotic susceptibility testing of carbapenem-resistant Enterobacteriaceae. PLoS Biol. 2020;18(3):e3000652 10.1371/journal.pbio.3000652 PMC7081982 32191697

[101] Banoo S, Bell D, Bossuyt P, Herring A, Mabey D, Poole F, et al Evaluation of diagnostic tests for infectious diseases: general principles. Nat Rev Microbiol. 2006;4(9 Suppl):S21–31. 10.1038/nrmicro1523 17034069

[102] CDC. CDC & FDA Antibiotic Resistance Isolate Bank: Neisseria gonorrhoeae. 2019.

[103] CLSI. M100-S25 Performance Standards for Antimicrobial Susceptibility Testing. CLSI. 2015;35(3).

[104] Sigma. Buffer Reference Center. Available from: https://www.sigmaaldrich.com/life-science/core-bioreagents/biological-buffers/learning-center/buffer-reference-center.html. [cited 2019 Jan 17]

[105] Lee SR, Chung JM, Kim YG. Rapid one step detection of pathogenic bacteria in urine with sexually transmitted disease (STD) and prostatitis patient by multiplex PCR assay (mPCR). J Microbiol. 2007;45(5):453–9. 17978806

[106] Kreutz JE, Munson T, Huynh T, Shen F, Du W, Ismagilov RF. Theoretical design and analysis of multivolume digital assays with wide dynamic range validated experimentally with microfluidic digital PCR. Anal Chem. 2011;83(21):8158–68. 10.1021/ac201658s 21981344 PMC3216679

