# Supplementary figures and images for: Surfactant-enhanced DNA accessibility to nuclease accelerates phenotypic β-lactam antibiotic susceptibility testing of Neisseria gonorrhoeae
Source: PLoS Biol. 2020 Mar 19;18(3):e3000651. doi: 10.1371/journal.pbio.3000651 (PMC7081974; doi:10.1371/journal.pbio.3000651)

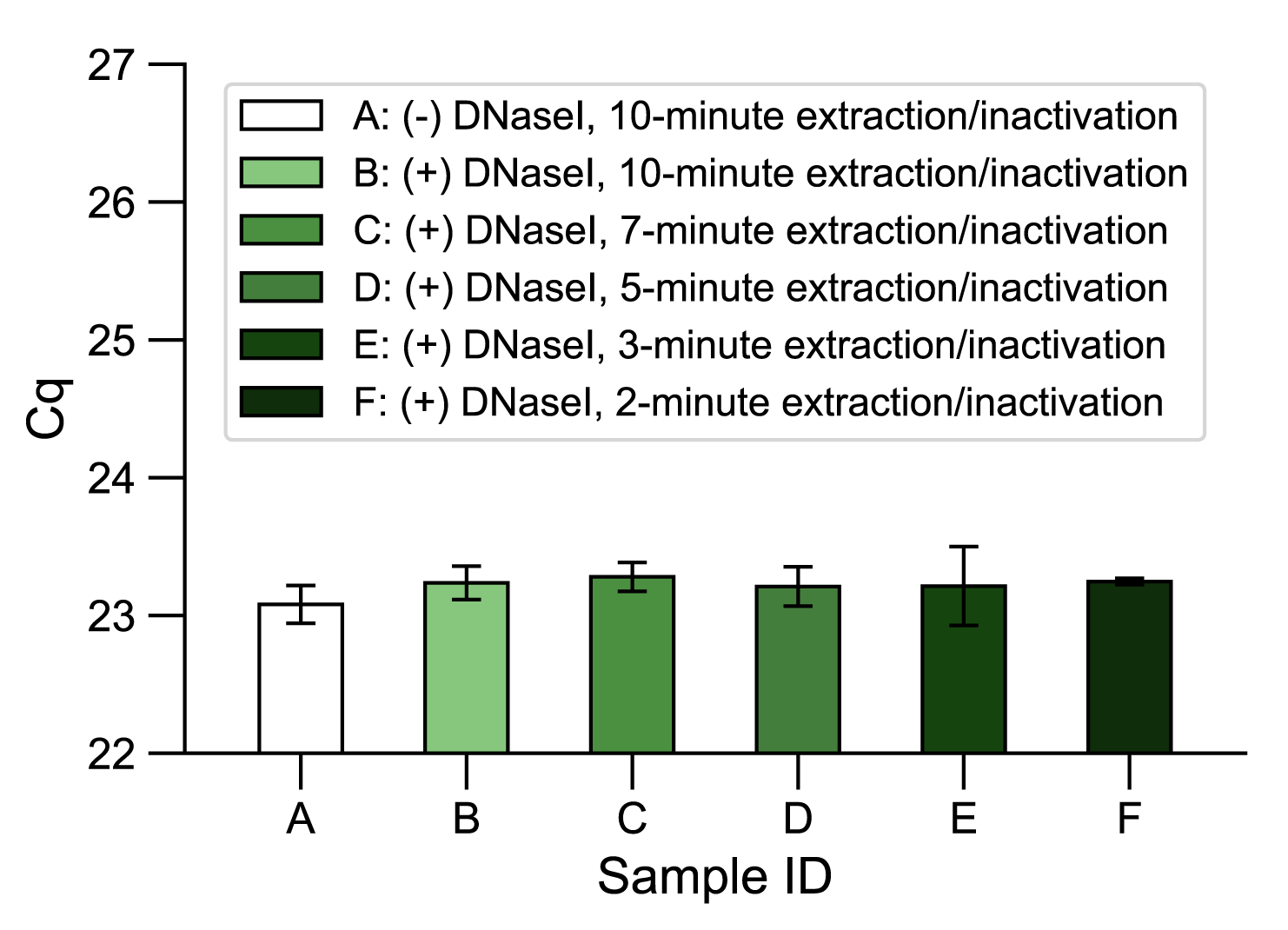

Supplement: S1 Fig — After extraction/inactivation, Ng DNA was spiked into the extractions containing the inactivated DNase I and incubated at 37°C. The Ng DNA was not degraded, confirming the inactivation of the DNase I enzyme. The concentration of DNase I, the composition of the incubations, and the extraction conditions were all performed under the same conditions as the ASTs. Error bars are 98% confidence intervals for three PCR replicates [55]. Data are in S8 Table. AST, antibiotic susceptibility test; Ng, N. gonorrhoeae. (TIF) [file pbio.3000651.s001.tif]

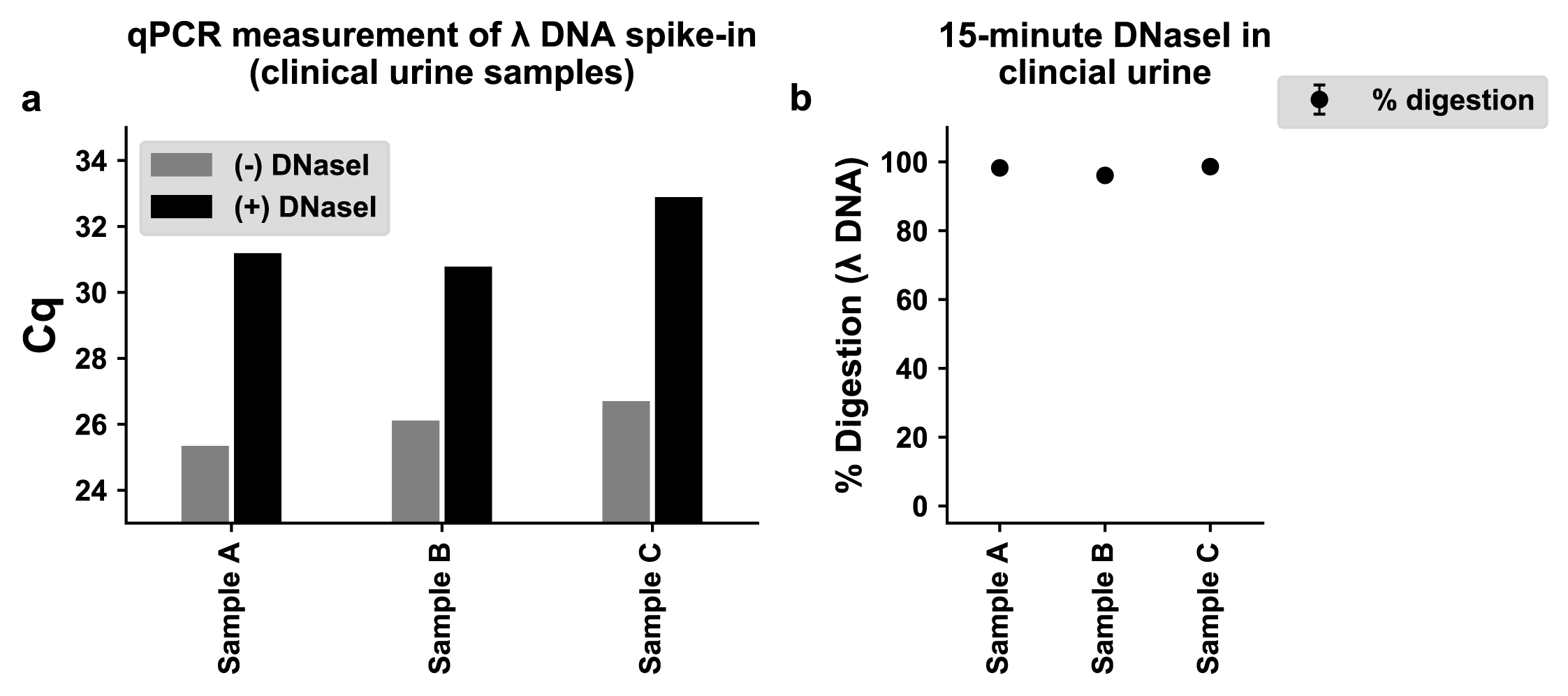

Supplement: S2 Fig — (a) The qPCR results for the lambda DNA spike-in for three different urine samples. (b) Percentage of DNA digested calculated from qPCR results with the same equations used previously (see Methods) to calculate percentage lysis. Data are in S9 Table. qPCR, quantitative PCR. (TIF) [file pbio.3000651.s002.tif]

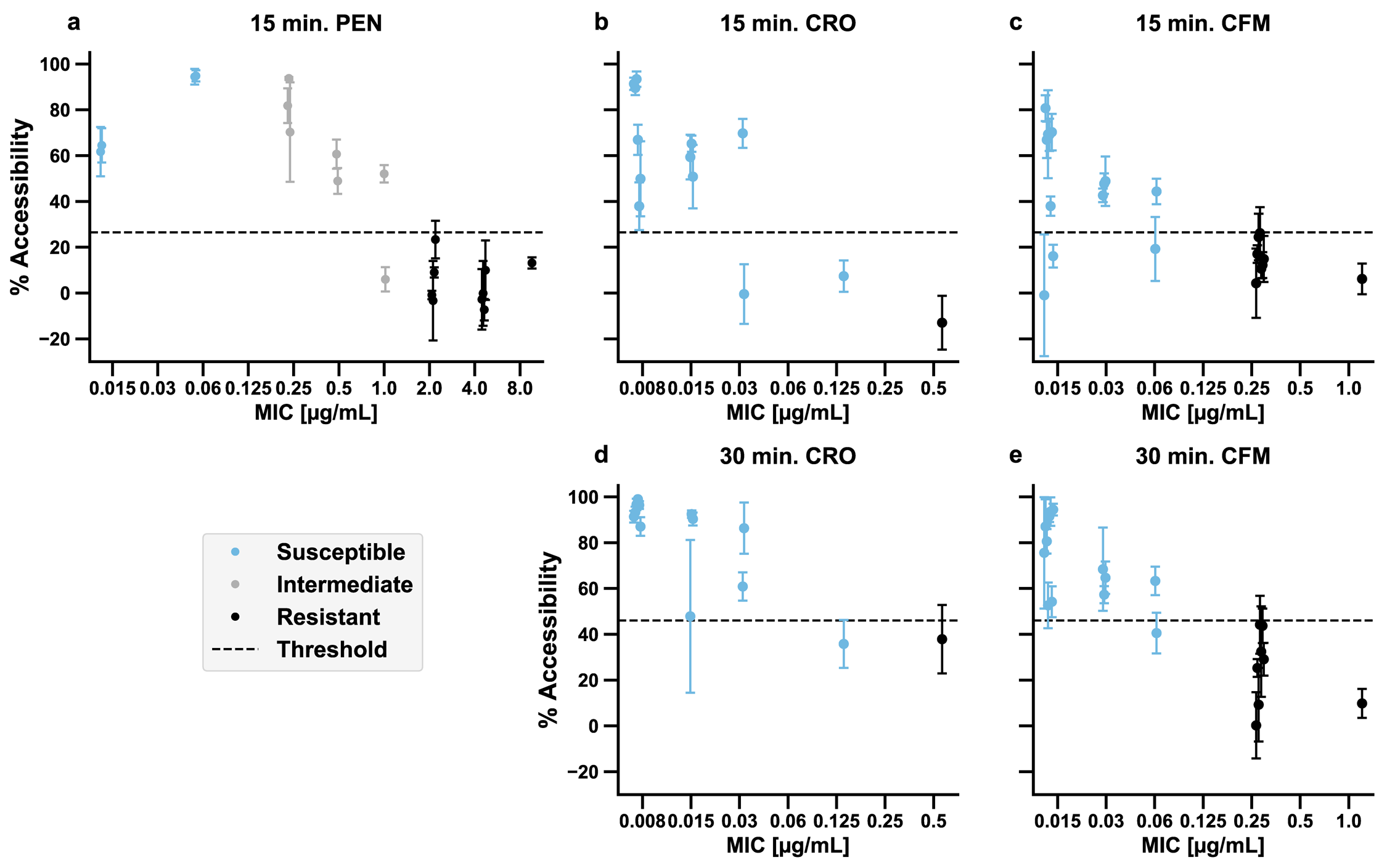

Supplement: S3 Fig — (a-c) Results of nuc-aAST after 15 min of exposure to (a) PEN, (b) CFM, or (c) CRO. (d-e) nuc-aAST results after exposure to (d) CFM or (e) CRO for 30 min. Each point represents the average for a single isolate run in (at least) biological triplicate for that condition; error bars represent the standard deviation from the biological replicates. All PCR assays were performed in technical triplicate. The dashed line represents the susceptibility threshold, which was set at 26.5% accessibility for 15-min exposures and 46% for 30-min exposures. Data are also plotted in Fig 4. (Data plotted here are in Table S4; experimental data from individual replicates are in S11 Table and S12 Table; MICs are in S1 Table). AST, antibiotic susceptibility test; CFM, cefixime; CRO, ceftriaxone; MIC, minimum inhibitory concentration; nuc-aAST, nuclease-accessibility AST; PEN, penicillin. (TIF) [file pbio.3000651.s003.tif]

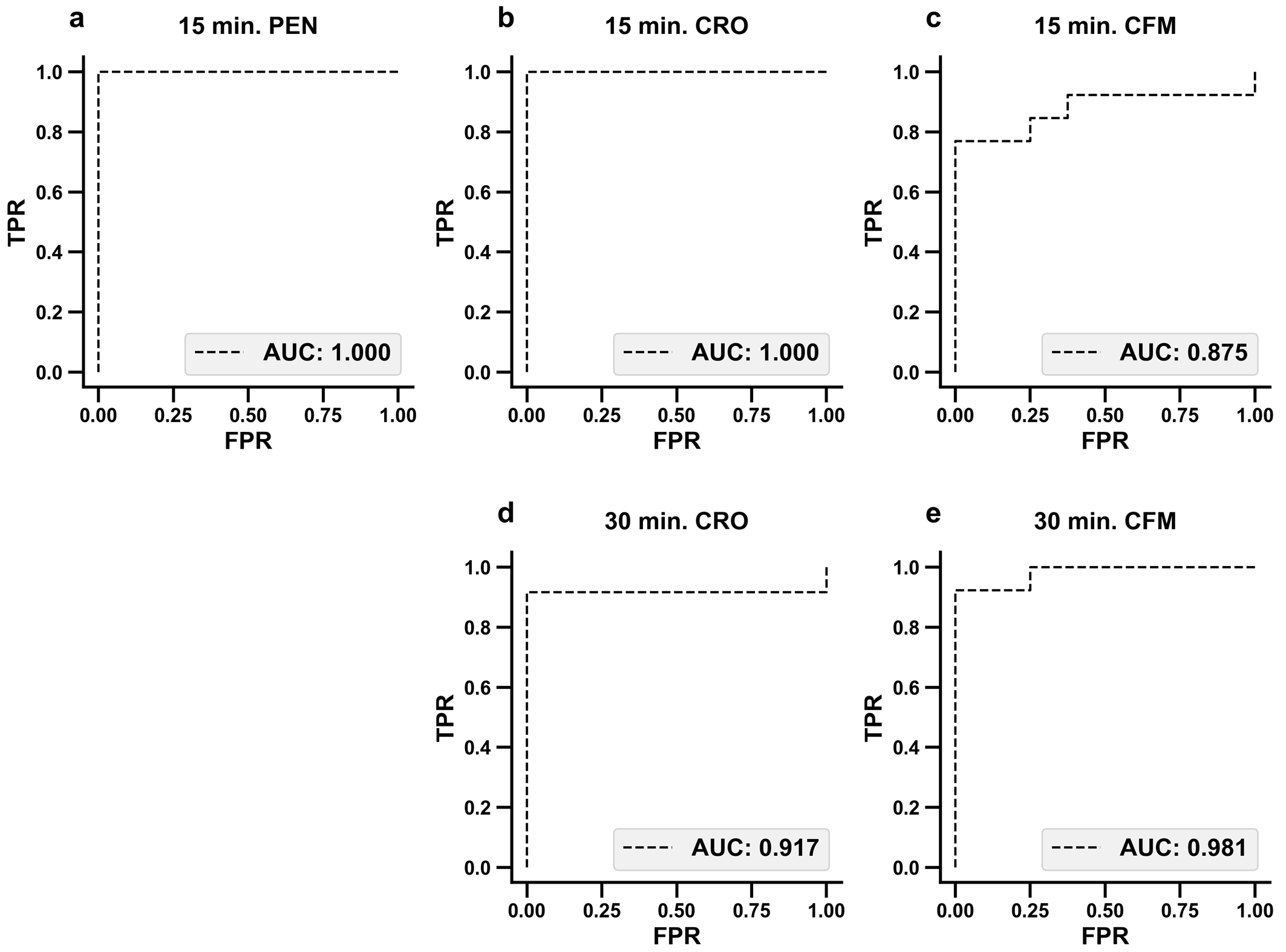

Supplement: S4 Fig — ROC curves were generated from the data shown in Fig 4, S4 Table. The FPR is shown on the x-axis and the TPR is shown on the y-axis. The AUC is shown for each plot. (a-c) Results of nuc-aAST after 15 min of exposure to (a) PEN, (b) CFM, or (c) CRO. (d-e) The nuc-aAST results after exposure to (d) CFM or (e) CRO for 30 min. AUC, area under the curve; CFM, cefixime; CRO, ceftriaxone; FPR, false positive rate; nuc-aAST, nuclease-accessibility antimicrobial susceptibility testing; PEN, penicillin; ROC, receiver operating characteristic; TPR, true positive rate. (TIF) [file pbio.3000651.s004.tif]
