# Supplementary material for: Surfactant-enhanced DNA accessibility to nuclease accelerates phenotypic β-lactam antibiotic susceptibility testing of Neisseria gonorrhoeae
Source: PLoS Biol. 2020 Mar 19;18(3):e3000651. doi: 10.1371/journal.pbio.3000651 (PMC7081974; doi:10.1371/journal.pbio.3000651)
Supplement: S1 Text — (PDF) [file pbio.3000651.s017.pdf]

Supplementary Materials and Methods

DNase I inactivation experiment. Incubations were performed in PCR tubes on a Bio-Rad C1000 Thermal Cycler. Samples including DNase I (Samples B-F in S1 Fig) consisted of 90 µL MHB, 5 µL $NaHCO_3$ (100 mM), and 5 µL NEB DNase I (2000 U/mL). Nuclease-free water was used in place of DNase I in the control sample (Sample A in S1 Fig). After vortexing to mix, 5 µL of each suspension was added to 45 µL QuickExtract DNA Extraction Solution (Lucigen). Samples A and B were heated at 65 °C for 6 min and 98 °C for 4 min. Sample C was heated at 65 °C for 4 min and 98 °C for 3 min. Sample D was heated at 65 °C for 3 min and 98 °C for 2 min. Sample E was heated at 65 °C for 2 min and 98 °C for 1 min. Sample F was heated at 65 °C for 1 min and 98 °C for 1 min. After each heating step was completed, the samples were placed on an ice block. DNase I activity was tested by adding 15 µL of a spike-in control of *Ng* DNA (1/20 dilution of stock Cq 19, expected concentration in PCR in this experiment to be a Cq of 23.3) to 15 µL of the Samples A-F in PCR tubes, samples were heated to 37 °C for 5 min, and then the samples were quantified in qPCR, as described in the main methods section.

DNA digestion of the lambda spike-in. Seven or eight 1.25 mL aliquots of clinical urine samples were centrifuged in 2 mL microcentrifuge tubes (VWR) for 5 min at 1,000 x g (Eppendorf 5418R). Pellets were re-suspended in 250 µL GWM containing a lambda DNA spike-in (see below for preparation). All working suspensions were then pooled together. To generate the (+) DNase I controls, 320 µL aliquots of the working suspensions were added to 40 µL 10X DNase I Reaction Buffer (NEB), 20 µL DNase I (2,000 U/mL) (NEB), and 20 µL nuclease-free water. Nuclease-free water was used in place of DNase I in the (-) DNase I control sample. Suspensions were then vortexed, quickly spun on a benchtop microcentrifuge (Labnet) at 2,000 x g for 2-3 sec, and incubated at 37 °C for 15 min on a heat block (ThermoScientific Digital Shaking Drybath). Suspensions were then removed from the heat block, vortexed, and spun on a benchtop microcentrifuge (Labnet) at 2,000 x g for 2-3 sec. After mixing, 20 µL of each suspension was added to 80 µL QuickExtract DNA Extraction Solution (Lucigen). Samples were heated at 65 °C for 6 min and 98 °C for 4 min on a BioRad C1000 Thermal Cycler. DNase I activity was tested by subtracting the concentration of lambda DNA in the (-) DNase I control from the concentration in the (+) DNase I control tube. Quantification of DNA was done with qPCR (Roche, LightCycler 96) as described in the main methods. As in the clinical nuc-aAST, we used 2 µL of template per 10 µL qPCR reaction.

The lambda DNA spike-in was prepared as follows. 12.5 µL lambda phage DNA ($7.1x10^5$ copies/µL, as quantified with ddPCR) (QX200, Bio-Rad) in 0.5X TE Buffer was mixed with 1238 µL Graver-Wade Medium (GWM).

Detailed statement of author contributions

ESS performed initial testing of osmotic, autolysis, and surfactant enhancers. ESS optimized sample handling prior to ABX exposure. ESS performed and analyzed enhancer testing and nuc-aAST experiments (Figs. 3, 4, 6), and assisted in performing digital LAMP experiments (Fig. 6). ESS performed data analysis and selected optimal conditions for nuc-aAST. ESS was a major contributor in selecting the readout metric of percent accessibility. ESS led experimental work and data analysis with clinical urine samples (Fig. 5d, 6e), and performed the experiments for the isolate replicates for (Fig. 5d). ESS contributed to writing the manuscript and figure design, created Fig. 5. and wrote the Methods section.

NGS guided initial testing of enhancers and developed two-step nuc-aAST workflow. NGS selected and performed initial screening of surfactant enhancers NGS optimized sample handling during ABX exposure. NGS performed and analyzed no-enhancer time course experiments (Fig. 2). NGS was a minor contributor in selecting the readout metric of percentage accessibility. NGS designed LAMP primers and contributed to the optimization of LAMP conditions for digital LAMP experiments (Fig. 6). NGS wrote the manuscript and created Figures 1-4, 6.

MMC performed experimental work and data analysis on the clinical urine samples (Fig. 5). MMC assisted in collecting the data for fig 4. MMC contributed to writing the Methods section.

JCR optimized digital LAMP conditions, and performed and analyzed all digital LAMP experiments for Fig. 6.

OOS provided isolates and guided discussion on gold-standard AST, and current treatment practices, and performed gold-standard agar dilution AST for *Ng*.

JDK coordinated and provided oversight of clinical-sample collection at AHF, provided technical assistance to AHF staff, and guided the selection of eligibility criteria for patient recruitment.
